# Beyond sleep duration: protocol for a systematic review of multidimensional sleep health in relation to cardiovascular disease and mortality

**DOI:** 10.3389/frsle.2024.1400562

**Published:** 2024-08-29

**Authors:** Mio Kobayashi Frisk, Daniil Lisik, Ding Zou

**Affiliations:** ^1^Center for Sleep and Vigilance Disorders, Institute of Medicine, University of Gothenburg, Gothenburg, Sweden; ^2^Krefting Research Centre, Institute of Medicine, Sahlgrenska Academy, University of Gothenburg, Gothenburg, Sweden

**Keywords:** circadian, lifestyle, longitudinal study, MACE, personalized medicine, preventive medicine, risk factor, sleep disorder

## Abstract

Adequate sleep duration has recently been recognized as a major determinant of cardiovascular health by the American Heart Association. This is a significant step toward recognizing sleep as a major lifestyle factor and pillar of health, along with physical activity and nutrition. However, healthy sleep is not only a matter of duration. Other dimensions, such as timing, regularity, efficiency, satisfaction with sleep, and daytime alertness are also deemed important to consider. We have designed a systematic review protocol according to the PRISMA-P guidelines with the objective of determining which sleep dimensions are predictors of all-cause mortality and major adverse cardiovascular events (MACE; cardiovascular death, non-fatal myocardial infarction, non-fatal stroke, and unstable angina requiring hospitalization), and whether or not the use of multiple dimensions of sleep yields superior predictive value to the use of sleep duration alone in predicting the above-mentioned outcomes. We will implement a systematic search strategy in 10 databases with independent manual screening by two reviewers. The aim is to comprehensively identify longitudinal studies which have examined the relationship between sleep duration and at least one other dimension of sleep and mortality or MACE. Meta-analysis will be performed after data extraction to address these objectives quantitatively. We anticipate that several sleep dimensions beyond sleep duration have been studied in relationship to all-cause mortality and MACE, and that a combination of multiple sleep dimensions can better predict these outcomes than sleep duration alone. Such findings would lay important groundwork to establish multidimensional sleep health as a major determinant of cardiovascular health.

## Introduction

Cardiovascular disease (CVD), especially major adverse cardiovascular events (MACE), which include cardiovascular death, non-fatal myocardial infarction, non-fatal stroke, and unstable angina requiring hospitalization (Bosco et al., 2021), is a leading cause of morbidity and mortality worldwide, with 523 million cases, and nearly 19 million deaths in 2019 (Roth et al., 2020). Primary prevention, with a focus on promoting health throughout the life course before manifest disease is a key strategy to combat the burden of CVD. In 2010, the American Heart Association (AHA) introduced a concept of “Cardiovascular Health” (CVH), which encompassed seven interrelated and modifiable health factors and behaviors, coined “Life's Simple Seven”(Lloyd-Jones et al., 2010). These were three lifestyle factors: diet, physical activity, and nicotine exposure; and four health factors: blood pressure, blood lipids, body weight, and blood glucose, which can be modified by lifestyle and targeted therapy. High CVH has been associated with lowered risk for not only CVD, but also cancer, end-stage renal disease and dementia, as well as many other benefits such as improved cognitive function and quality of life (Rasmussen-Torvik et al., 2013; Han et al., 2016; Virani et al., 2021).

Sleep was not included in the initial “Life's Simple Seven”, despite its influence on CVH as well as general health and wellbeing. The AHA updated their concept of CVH in 2022 to include sleep duration as the eighth factor of CVH, now termed “Life's Essential Eight” (Lloyd-Jones et al., 2022). Sleep duration is indeed a major health factor, demonstrated by evidence that both short and long sleep duration is associated with increased cardiovascular events and all-cause mortality (Wingard and Berkman, 1983; Yin et al., 2017). However, it is far from being the only sleep-related factor that is relevant to health.

Sleep is a physiological and behavioral state characterized by a lack of consciousness and voluntary movement and considered a period of rest and recovery in which humans spend about one third of their lives (Aminoff et al., 2011). It is a physiologically complex state that is essential for wellbeing, health, and even survival, as can be demonstrated by the ultimately lethal effects of sleep deprivation (Everson et al., 1989). Although yet to be fully clarified, sleep is known to be involved in childhood development (Roffwarg et al., 1966; Mirmiran et al., 1983; Zielinski et al., 2016), energy conservation (Benington and Heller, 1995; Scharf et al., 2008; Zielinski et al., 2016), immune modulation (Krueger, 2008; Zielinski and Krueger, 2011; Zielinski et al., 2016), cognitive and physical performance (Walker, 2008; Killgore, 2010; Zielinski et al., 2016; Watson, 2017; Cunha et al., 2023), psychological wellbeing (Scott et al., 2021), and clearance of brain waste (Xie et al., 2013; Zielinski et al., 2016).

There are numerous ways to characterize and evaluate sleep, and many factors are involved in the construct of “healthy sleep”. Polysomnography, for instance, can be used to study sleep architecture and duration, as well as to identify sleep-related disorders such as sleep-disordered breathing, periodic limb movements, and narcolepsy. Actigraphy and sleep diaries used over an extended period of time can be used to identify rest-activity patterns including sleep duration, timing, and regularity as well as environmental factors. Questionnaires and interviews can be used to determine how individuals perceive their sleep, as well as to evaluate daytime symptoms such as fatigue and sleepiness. Neuroimaging with positron emission tomography (PET), single-photon emission computed tomography (SPECT), and functional magnetic resonance imaging (fMRI) have been used in research to study metabolism, cerebral blood flow, waste build-up, and neurotransmission in sleep (Dang-Vu et al., 2010; Pak et al., 2020). Many of these individual sleep factors, including perceived quality, timing, latency, duration, daytime alertness, napping, and sleep disorders have been associated with mortality, morbidity and wellbeing, suggesting that it is relevant to consider multiple factors in the assessment of sleep as a component of CVH and a major pillar of healthy lifestyle (Kojima et al., 2000; Newman et al., 2000; Akerstedt et al., 2004; Jennings et al., 2007; Young et al., 2008; Gottlieb et al., 2010; Laugsand et al., 2011; Ohayon et al., 2014; Cubo et al., 2019; Zhang et al., 2019).

There currently exist several tools which measure sleep health as a complex construct, taking multiple dimensions into account. Buysse's “RU-SATED” scale, for example, rates sleep regularity, sleep satisfaction, daytime alertness, sleep timing, sleep efficiency, and sleep duration to evaluate sleep health (Buysse, 2014). A “healthy sleep scale” (HSS), which combines sleep duration, chronotype, insomnia, snoring, and excessive daytime sleepiness to evaluate sleep health has been developed in the UK biobank cohort, and shown to predict CVD in several studies (Fan et al., 2020; Wang et al., 2022; Nambiema et al., 2023). The National Sleep Foundation has developed a “Sleep Health Index” (SHI) which uses 14 questions to evaluate sleep duration, sleep disorders, and sleep quality (Knutson et al., 2017). “Sleep quality” is a general term which can be considered a composite of several sleep-related factors; in the SHI this refers to feelings of being well-rested, trouble falling asleep and staying asleep, negative impact due to lack of sleep, and unintentional dozing. The “Pittsburgh Sleep Quality Index” (PSQI), also developed by Buysse and colleagues, is one of the most commonly used tools to assess “sleep quality”. The PSQI consists of nine questions evaluating bedtime, time to fall asleep, time one gets out of bed, sleep duration and time in bed, trouble sleeping due to several factors, use of sleep medications, trouble staying awake, enthusiasm to get things done, and a global rating of sleep quality (Buysse et al., 1989). As such, it can also be considered a tool to measure multidimensional sleep health. Yin et. al.'s “Sleep Quality Scale”(Yi et al., 2006), Partinen and Gilason's “Basic Nordic Sleep Questionnaire”(Partinen and Gislason, 1995) are other such questionnaires. Poor sleep health as evaluated by tools measuring multidimensional sleep have been associated with stress (Benham, 2019), CVD (Brindle et al., 2019; Fan et al., 2020; Lee et al., 2022; Wang et al., 2022; Nambiema et al., 2023; Tian et al., 2023), and mortality (Lee et al., 2023).

Current evidence thus suggests that multiple dimensions of sleep health should be considered to achieve optimal health and wellbeing, rather than the AHAs current recommendation of sleep duration alone (Lloyd-Jones et al., 2022). Synthesis of evidence, however, is currently lacking. In this systematic review, we will summarize longitudinal observational studies which have examined the relationship between all-cause mortality and/or MACE and sleep duration in addition to at least one other sleep health factor. Subsequently, we plan to describe which sleep health factors have been studied and associated with the given endpoints. A meta-analysis will be conducted to quantitatively synthesize comparable numerical data (Lisik et al., 2023), in order to determine which factors are most influential in predicting all-cause mortality and/or MACE, if a composite of multiple factors improves predictive capacity of the endpoints, and how many factors should ideally be considered.

## Methods

### Reporting and protocol registration

This protocol was prepared according to the Preferred Reporting Items for Systematic Reviews and Meta-Analyses Protocols (PRISMA-P) (Shamseer et al., 2015). It has been registered with the International Prospective Register of Systematic Reviews (PROSPERO) with the title, *Beyond sleep duration: a systematic review of multidimensional sleep health in relation to cardiovascular disease and mortality* and registration number CRD42024503231. The final report will be written according to the Preferred Reporting Items for Systematic Reviews and Meta-Analyses (PRISMA) guidelines (Page et al., 2021), and any deviations from the original protocol will be reported in the final manuscript.

### Inclusion/exclusion criteria and outcome measures

This review will include observational longitudinal follow-up studies of adults ≥18 years at the start of follow-up. The studies must include an assessment of the participants' sleep duration as well as at least one other sleep dimension (such as sleep timing, regularity, efficiency, daytime alertness and napping, circadian factors, and sleep disorders), as well as MACE and/or death as an endpoint. The outcome measure in this systematic review will be MACE, which in this context will refer to cardiovascular death, non-fatal myocardial infarction, non-fatal stroke, and unstable angina requiring hospitalization, as well as all-cause mortality (Bosco et al., 2021). There will be no exclusion due to language. Google Translate will be used to translate all non-English language reports (Jackson et al., 2019).

### Research questions

We aim to answer the following questions:

1. Which sleep health dimensions have been combined with sleep duration to assess MACE and all-cause mortality?
2. Does combining multiple dimensions of sleep provide an added value for predicting MACE and all-cause mortality?
  a. Which sleep dimensions are predictors of MACE and all-cause mortality?
  b. Does combination of a greater number of sleep dimensions result in better prediction of the endpoints?

### Search strategy

Bibliographic database searches were performed on January 22nd 2024 in 10 databases (CAB Direct, CINAHL, Embase, Google Scholar, PsycINFO, PubMed, Scopus, Web of Science, WHO Global Index Medicus, and WorldCat Dissertations and Theses), with search queries tailored to each database due to differences in syntax and availability of controlled vocabulary.

Table 1 illustrates the search strategy for PubMed, while search queries for the other databases can be found in the Supplementary material.

**Table 1:** Search strategy for PubMed.

**#**	**Block name**	**Search terms**
**1**	Sleep duration	**(** (“Sleep Duration”[mh] OR “Sleep Deprivation”[mh] OR “sleep deprivation”[tiab] OR “insufficient sleep”[tiab]) OR (“quantity sleep”[tiab:~2] OR “quantities sleep”[tiab:~2] OR “amount sleep”[tiab:~2] OR “duration sleep”[tiab:~2] OR “length sleep”[tiab:~2] OR “time sleep”[tiab:~2] OR “period sleep”[tiab:~2] OR “hours sleep”[tiab:~2] OR “minutes sleep”[tiab:~2] OR “span sleep”[tiab:~2]) OR (“duration asleep”[tiab:~2] OR “length asleep”[tiab:~2] OR “time asleep”[tiab:~2] OR “period asleep”[tiab:~2] OR “hours asleep”[tiab:~2] OR “minutes asleep”[tiab:~2]) OR (“quantity sleeping”[tiab:~2] OR “quantities sleeping”[tiab:~2] OR “amount sleeping”[tiab:~2] OR “duration sleeping”[tiab:~2] OR “length sleeping”[tiab:~2] OR “time sleeping”[tiab:~2] OR “period sleeping”[tiab:~2] OR “hours sleeping”[tiab:~2] OR “minutes sleeping”[tiab:~2] OR “span sleeping”[tiab:~2]) OR (“short sleep”[tiab:~2] OR “long sleep”[tiab:~2] OR “extended sleep”[tiab:~2]) OR (“short sleeper”[tiab:~2] OR “long sleeper”[tiab:~2] OR “extended sleeper”[tiab:~2]) OR (“short sleepers”[tiab:~2] OR “long sleepers”[tiab:~2] OR “extended sleepers”[tiab:~2]) **)**
**2**	Sleep components	**(** (“Sleep”[mh] OR $\text{“slee}{\text{p}}^{*}\text{”[tiab]}$ OR $\text{“wak}{\text{e}}^{\text{*}}\text{”[tiab]}$ OR “waking”[tiab] OR “awake”[tiab]) OR (“Polysomnography”[mh] OR “Actigraphy”[mh] OR “actigraph*”[tiab] OR “actimetr*”[tiab] OR “acceleromet*”[tiab] OR “polysomnograph*”[tiab] OR “EEG”[tiab] OR “electroencephalogram”[tiab] OR “MSLT”[tiab] OR “MWT”[tiab] OR “fitbit”[tiab] OR “dreem”[tiab] OR “Oura ring”[tiab] OR “Gen3”[tiab] OR “Fitbit”[tiab] OR “Mi band”[tiab]) OR (“Circadian Clocks”[mh] OR “Circadian Rhythm”[mh] OR “circadian”[tiab] OR “chronotype*”[tiab] OR “chronotherap*”[tiab] OR “eveningness”[tiab] OR “morningness”[tiab] OR “evening type*”[tiab] OR “morning type*”[tiab] OR “bedtime*”[tiab] OR “time to bed”[tiab] OR “time in bed”[tiab] OR “shuteye”[tiab] OR “shut-eye”[tiab] OR “lights off”[tiab] OR “lights on”[tiab] OR “Shift Work Schedule”[mh] OR “shift work*”[tiab] OR “shift work*”[tiab] OR “shift schedule”[tiab:~2] OR “shift schedules”[tiab:~2] OR “shift scheduling”[tiab:~2] OR “shifting schedule”[tiab:~2] OR “shifting schedules”[tiab:~2] OR “shifting scheduling”[tiab:~2] OR “working hours”[tiab:~2] OR “work hours”[tiab:~2] OR “work schedule”[tiab:~2] OR “work schedules”[tiab:~2] OR “work scheduling”[tiab:~2] OR “working schedule”[tiab:~2] OR “working schedules”[tiab:~2] OR “jetlag”[tiab] OR “jet-lag”[tiab] OR “light*”[tiab] OR “nois*”[tiab] OR “WASO”[tiab] OR “TIB”[tiab] OR “SE”[tiab]) OR (“Sleepiness”[mh] OR “fatigue*”[tiab] OR “tired*”[tiab] OR “somnolence”[tiab] OR “nap”[tiab] OR “napping”[tiab] OR “alert*”[tiab] OR “ESS”[tiab] OR “KSS”[tiab] OR “EDS”[tiab] OR “day”[tiab] OR “daytime”[tiab] OR “night*”[tiab] OR “drows*”[tiab] OR “siesta”[tiab]) OR (“Sleep Wake Disorders”[mh] OR “insomnia”[tiab] OR “restless legs syndrome”[tiab] OR “restless leg syndrome”[tiab] OR “Willis-Ekbom”[tiab] OR “Wittmaack-Ekbom”[tiab] OR “RLS”[tiab] OR “periodic leg movement*”[tiab] OR “periodic limb movement*”[tiab] OR “Snoring”[mh] OR “snoring”[tiab] OR “snore”[tiab] OR “hypersomnia”[tiab] OR “dyssomnia”[tiab] OR “parasomnia”[tiab] OR “narcolepsy”[tiab] OR “night terror”[tiab] OR “nightmare*”[tiab] OR “Apnea”[mh] OR “apnea”[tiab] OR “apnoea”[tiab] OR “hypopnea”[tiab] OR “hypopnea”[tiab] OR “OSA”[tiab] OR “OSAHS”[tiab] OR “AHI”[tiab] OR “CSA”[tiab] OR “UARS”[tiab] OR “upper airway resistance syndrome”[tiab]) **)**
**3**	Multidimensionality	**(** (“Sleep”[mh] OR “sleep*”[tiab]) AND ( (“RU-SATED”[tiab] OR “RU_SATED”[tiab] OR “PSQI”[tiab]) OR (“score*”[tiab] OR “index”[tiab] OR “indices”[tiab] OR “multidimensional”[tiab] OR “multi-dimensional”[tiab] OR “multi*”[tiab] OR “dimension*”[tiab] OR “component*”[tiab] OR “parameter*”[tiab] OR “metric*”[tiab] OR “composite”[tiab] OR “combination*”[tiab]) ) **)**
**4**	Multidimensional sleep health	**(#1** AND **(#2** OR **#3))**
**5**	All-cause outcomes	**(**“Mortality”[mh] OR “mortality”[tiab] OR “death*”[tiab] OR “lethal”[tiab]**)**
**6**	Specific outcomes of interest	**(** (“Heart Arrest”[mh] OR “SCD”[tiab] OR “cardiopulmonary arrest”[tiab] OR “cardiac arrest”[tiab] OR “heart arrest”[tiab] OR “asystole”[tiab] OR “cardiac event*”[tiab] OR “Myocardial Infarction”[mh] OR “myocardial infarct*”[tiab] OR “Myocardial Ischemia”[mh] OR “myocardial ischemia”[tiab] OR “unstable angina”[tiab] OR “acute coronary syndrome”[tiab] OR “ACS”[tiab] OR “AMI”[tiab] OR “MI”[tiab] OR “Heart Failure”[mh] OR “heart failure”[tiab] OR “cardiac failure”[tiab] OR “myocardial failure”[tiab] OR “heart decompensation”[tiab] OR “ventricular dysfunction”[tiab] OR “CHF”[tiab]) OR (“Stroke”[mh] OR “stroke”[tiab] OR “cerebral infarct*”[tiab] OR “cerebrovascular accident*”[tiab] OR “CVA”[tiab] OR “brain vascular accident*”[tiab] OR “cerebrovascular apoplexy”[tiab] OR “brain ischemia”[tiab] OR “intracranial hemorrhage”[tiab] OR “intracranial hemorrhage”[tiab] OR “cerebral hemorrhage”[tiab] OR “cerebral hemorrhage”[tiab]) OR (“MACE”[tiab] OR “major adverse cardiovascular event*”[tiab] OR “infarct*”[tiab]) **)**
**7**	Outcomes	**(**#5 OR #6**)**
**8**	Full query	**#4** AND **#7**

mh, search to be made by Medical Subject Headings (MeSH), a controlled vocabulary; tiab, search to be made in title and abstract fields [search terms followed by “:~2” in the field setting denote search terms with a proximity parameter (in which the two words are allowed to occur in any order with up to two words in-between)]. **Clarifications. (**1) **bold font-weight** visually indicates blocks of search terms or parentheses encapsulating these; (2) green color denotes blocks of search terms; (3) dark blue color denotes search terms with the [tiab] field setting without the proximity parameter; (4) light blue color denotes search terms with the [tiab] field setting with the proximity parameter; (5) purple color denotes controlled vocabulary search terms.

The search terms were based on domain knowledge and were refined/extended with pilot searches. Where possible, relevant controlled vocabulary terms were included. Spelling and tense variations were accounted for. This general search strategy was used for nine databases, while it was simplified for Google Scholar due to substantial search limitations in this database.

### De-duplication and screening

De-duplication will be conducted in Endnote 21 (Clarivate Analytics, 2023), as previously described by Bramer et al. (2016). Thereafter, an initial screening will be performed based on title, abstract, and keywords. Records which are clearly eligible or for which there is doubt about eligibility will pass to the second step. In the second step, the full text of each record will be retrieved and assessed for eligibility. Both steps of the screening will be performed independently by two reviewers (MKF and DZ), blinded to each other's decisions during each step. Decisions will be unblinded and compared for differences after each step. Disagreements will be resolved through discussion and if needed through arbitration by a third reviewer (DL). Rayyan (rayyan.ai) will be used for screening and documentation of decisions. The main reason for exclusion at the second screening step will be presented in a Supplementary Table in the final manuscript.

### Data extraction

Data to be extracted from the included articles are: surname of first author, study design, country, number of subjects and subject characteristics (age, sex, and comorbidities), sleep duration, other sleep-related parameters, outcome definition, outcome data (point estimates and corresponding 95%CI), and length of follow-up. A standardized Microsoft Excel (Microsoft Corp., 2024) form will be used to extract the data, and corresponding authors will be contacted in order to obtain any missing data. Two reviewers (MKF and DZ) will independently perform all data extraction blinded to the other reviewer's work. Differences will be compared and discussed after completion with arbitration by a third reviewer (DL) where necessary.

### Assessment of quality and risk of bias

The Newcastle-Ottawa Quality Rating Scale (NOS) will be used to assess the quality and risk of bias of included articles (Wells et al., 2021). NOS assesses selection of study participants, comparability of cohorts, and the outcome, and is a commonly and easily used quality assessment tool for cohort studies (Deeks et al., 2003; Higgins, 2008; Ma et al., 2020). Two reviewers (MKF and DZ) will independently assess the articles and any disagreement will be resolved by consensus. If needed, a third reviewer (DL) will arbitrate the final rating.

### Data synthesis and statistical analysis

Extracted data items will be summarized in a table of characteristics. In addition, relevant aspects will be synthesized narratively. Comparable data (defined here as results from regression analysis with sufficient similarity in study participants, exposure, and outcome, as assessed by MKF and DZ) will be quantitively synthesized using random-effects meta-analysis with robust variance estimation (RVE) (Hedges et al., 2010). The random-effects model has been chosen as it is expected that the included studies will demonstrate substantial heterogeneity, given different cohorts, exposure definitions/assessment methods, outcome definitions/assessment methods, as well as statistical analysis approaches. The RVE model will be utilized as it relaxes a number of assumptions of conventional methods, such as normal distribution of effect sizes and their estimates. Furthermore, RVE can accommodate non-independent effect sizes (Pustejovsky and Tipton, 2022). As it is expected that some included studies may investigate multiple combinations of sleep health dimensions (and reuse controls), RVE will enable the inclusion of all such effect sizes in more comprehensive meta-analyses. A further strength of RVE is that the precise dependency structure (or degree) does not need to be defined in the model. RVE will be implemented through the *robumeta* R package (Fisher et al., 2015).

A meta-analysis will be performed for each distinct exposure-outcome pair, for which there are at least two studies with comparable numerical data (Ahn and Kang, 2018). Meta-analyses will be performed to assess the predictive value on all-cause mortality and/or MACE of:

1) Specific sleep health dimensions (and combinations thereof).
2) Quantity of sleep health dimensions.

If data allows, meta-regression will be performed to control for relevant confounders such as sex, age, body mass index, hypertension and diabetes.

**Figure 1:**
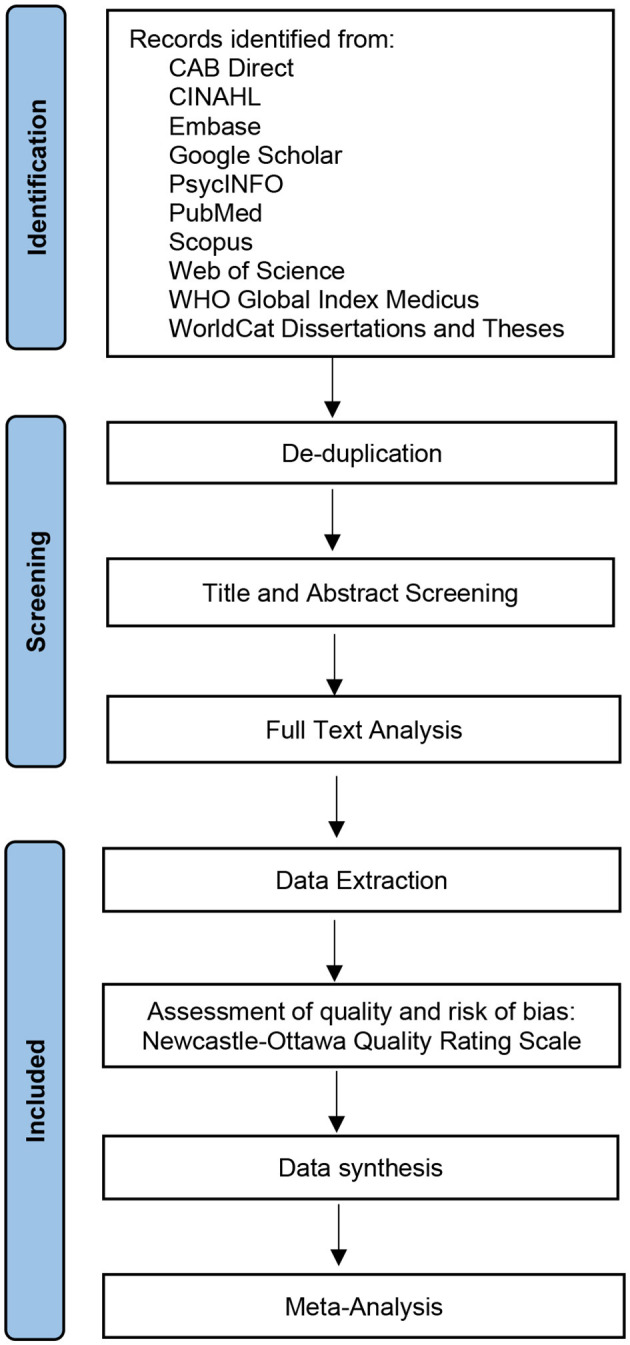
Schematic of review methods. *Modification of flow-chart based on:* Page et al. (2021). For more information, visit: http://www.prisma-statement.org/ .

Depending on the study characteristics (particularly if potential dependency in effect sizes is primarily due to common features of the researchers or assessment tools, or of the subjects), the appropriate weighting model will be chosen (Pustejovsky and Tipton, 2022). All meta-analyses will be performed with small sample adjustment for both the residuals and the degrees of freedom, as per the general recommendations and in particular given the expectation of relatively small numbers of studies in each meta-analysis (Tipton, 2015). A forest plot will be produced to visualize the results of each meta-analysis, using the *forestploter* R package (Dayimu, 2022). Heterogeneity will be assessed by the I-squared statistic (*I*^2^; to quantify the proportion of variation between studies not due to random sampling error) (Higgins et al., 2003) and Tau-squared (τ^2^; to determine the between-study variance of true effect) (Parr et al., 2019). In meta-analyses with Satterthwaite degrees of freedom (*df*_S_) below 4, the threshold for significant *p-*value will be 0.01 instead of the default level of 0.05 to reduce the risk of type I error (Tanner-Smith et al., 2016). Publication bias will be assessed in exposure-outcome pairs with ≥10 studies (Dalton et al., 2016) through statistical tests (Begg and Mazumdar correlation test and Egger's regression test, respectively, with *p-value* of 0.05 as the threshold for significance) and visual inspection of funnel plots. In case of suspected publication bias, we will estimate the number of effect sizes required to return symmetry using the trim-and-fill method. Assessment of publication bias will be undertaken using the *metafor* R package (Viechtbauer, n.d.). A sensitivity analysis excluding studies with a poor overall quality rating will be performed where at least two studies with comparable numerical data remain, to assess the influence of methodological rigor on the pooled results. Other secondary analyses which may be performed include subgroup analyses based on diagnosed sleep disorders, follow-up time, sex and age, if data allows as per above, to discern the stability of the association or potential cause(s) of heterogeneity. Analyses will be performed using the R statistical software (R Core Team, 2024). All data and code will be made available on Open Science Framework or as Supplementary material. The methods are outlined graphically in Figure 1, based on the PRISMA flow diagram layout (Page et al., 2021).

## Discussion

Sleep, an essential and intricate component of human life, is undergoing a transformative reevaluation in the realm of medical science. Traditionally, sleep medicine has centered on diagnosing and treating specific sleep disorders. However, there is a discernible shift toward recognizing sleep health as a comprehensive construct, encompassing various dimensions that collectively influence overall wellbeing (Buysse, 2014). This systematic review aims to explore the intricate relationship between multiple dimensions of sleep and their correlation with mortality and MACE, marking a groundbreaking step in understanding the broader implications of sleep on CVH.

The contemporary societal landscape, characterized by rapid technological advancements and evolving work structures, poses unprecedented challenges to our sleep patterns. Factors such as rigid work schedules, constant exposure to information, and transmeridian travel have become intrinsic disruptors of sleep. Artificial lighting, a product of technological progress, has detached humans from their natural rhythm dictated by the sun, introducing disruptions across various facets of sleep (Zhong et al., 2022). The past decade, marked by a surge in technology, has exacerbated this phenomenon by introducing devices that emit both light and distractions, further hindering the attainment of optimal sleep.

Temporal aspects of sleep have gained significant attention in recent research. Surprisingly, the timing of sleep initiation, with evidence suggesting that going to bed before 10 pm or after 11 pm is associated with poorer CVH than sleeping between 10 and 11 pm, irrespective of sleep duration, underscores the importance of circadian rhythms (Nikbakhtian et al., 2021). Even subjective factors like dissatisfaction with sleep (Del Brutto et al., 2024) and ease of falling asleep (Li et al., 2021) have shown associations with increased risk of mortality, emphasizing the need to delve beyond mere sleep duration. Recent findings highlight sleep regularity as a more accurate predictor of all-cause mortality than sleep duration alone (Cribb et al., 2023; Windred et al., 2024). This shift in focus toward regularity underscores the intricate interplay of sleep dimensions and their collective impact on health outcomes. Aspects such as consistency in sleep patterns, beyond the conventional consideration of sleep duration, are proving to be pivotal in understanding the complex relationship between sleep and mortality/MACE.

**Figure 2:**
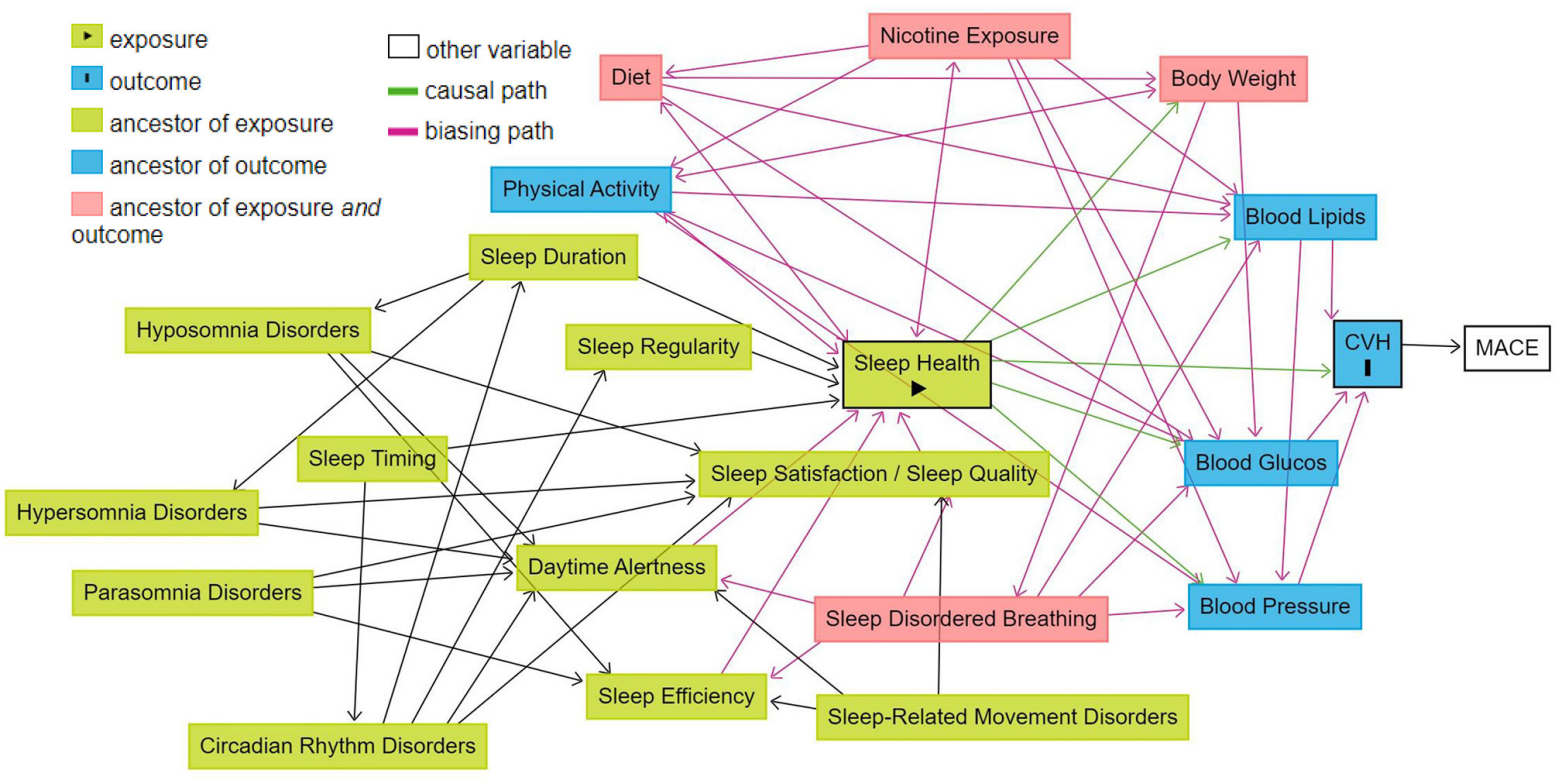
Schematic of the relationship between sleep health and cardiovascular health in relation to other health and life-style factors. Illustration of the causal relationships between fundamental sleep health factors, sleep disorders, the health and lifestyle factors included in the AHA's “simple seven”, CVH, and MACE. Created using the dagitty browser application: Textor et al. (2016).

Health, be it sleep health, CVH, or overall general health is a construct that is difficult to define in a straight-forward manner. Rather it, is the result of the complex interplay of multiple social/behavioral, environmental, and biological factors (Grandner and Fernandez, 2021). The impact of lifestyle factors such as physical activity (You, 2024), nutrition (You et al., 2024b), and sleep (You et al., 2024a) on overall health is critical for a holistic understanding of sleep duration. Figure 2 portrays a schematic which illustrates the causal relationships between fundamental sleep health factors, sleep disorders, the health and lifestyle factors included in the AHA's “simple seven”(Lloyd-Jones et al., 2010), CVH, and MACE, created using the DAGitty browser application (Textor et al., 2016). The directed edges (denoting a causal relation between two factors with specified direction) in the schematic have strong support in the literature (Scheer et al., 2009; Calhoun and Harding, 2010; Chennaoui et al., 2015; Visseren et al., 2021; Russell et al., 2023). The schematic also makes apparent one of the principal challenges that accompany the study of something as complex as health; the determination of causation. The studies we will include are observational, thus will limit the ability to assess the extent to which the studied sleep health factors independently cause MACE or mortality, both due to expected limitations in data to perform robust analyses and due to unobserved confounders (as exemplified by the intermediate processes such as hypertension). Moreover, as can be seen in the schematic, sleep disordered breathing has a particular relationship with other factors of cardiovascular health that is not shared with other sleep disorders due to the non-sleep-mediated effects of intermittent hypoxia on the cardiovascular system (Cowie et al., 2021). Such a relationship can further complicate the interpretation of our results, warranting a deeper investigation of the mechanisms linking various sleep factors to MACE, which is beyond the scope if this review. If meta-regression can be performed, this will allow us to determine whether there are underlying cardiovascular risk factors which can also explain the relationship between sleep factors and MACE.

Despite the aforementioned challenges, in an era defined by technological advancements and diverse lifestyles, recognizing and addressing the multifaceted nature of sleep is not only pertinent but may also pave the way for innovative preventive strategies against the growing burden of CVD. Anticipating that various sleep dimensions are intertwined with sleep duration in influencing mortality and MACE, this systematic review intends to contribute to the burgeoning field of multidimensional sleep health. The hypothesis posits that incorporating multiple sleep dimensions in longitudinal studies will yield superior predictive accuracy compared to a singular focus on sleep duration. Should this hypothesis be validated, it could pave the way for a paradigm shift in conceptualizing sleep health as a major determinant of CVH. Recognizing the heterogeneity of contemporary society, the call for a broad perspective on multidimensional sleep health is imperative. Tailoring interventions to individual physiologies and lifestyles becomes crucial in optimizing overall health outcomes. Moreover, since many sleep dimensions, in addition to being modifiable, are easily detectable by self-observation or with the help of readily available tools such as sleep diaries and smartphone applications (Fino and Mazzetti, 2019) there is a large potential for developing preventative strategies against the burden of CVD. On the individual level, this may be achieved by lifestyle modification to improve sleep health as well as to fine-tune risk prediction to identify individuals in need of more intensive preventive care. Such a strategy would open avenues for personalized interventions that go beyond treating sleep disorders to proactively promoting CVH through holistic sleep management. On the societal level, a deeper understanding of the relationship between sleep health and CVH would be instrumental in guiding work and school policy to optimize scheduling and environmental conditions. A synthesis of the existing evidence on the relationship between multiple sleep factors and hard cardiovascular endpoints will provide an important steppingstone for the development of ideal lifestyle-related health management strategies.

## Data Availability

The original contributions presented in the study are included in the article/Supplementary material, further inquiries can be directed to the corresponding author/s.
