# Supplementary material for: Beyond sleep duration: protocol for a systematic review of multidimensional sleep health in relation to cardiovascular disease and mortality
Source: Front Sleep. 2024 Aug 29;3:1400562. doi: 10.3389/frsle.2024.1400562 (PMC12713809; doi:10.3389/frsle.2024.1400562)
Supplement: Supplementary file 1 [file Table_1.DOCX]

Beyond sleep duration: protocol for a systematic review of multidimensional sleep health in relation to cardiovascular disease and mortality

Supplementary Material

Mio Kobayashi Frisk^1^, Daniil Lisik^2^, Ding Zou^1^

^1^Center for Sleep and Vigilance Disorders, Institute of Medicine, University of Gothenburg, Gothenburg, Sweden

^2^Krefting Research Centre, Institute of Medicine, Sahlgrenska Academy, University of Gothenburg, Gothenburg, Sweden

Correspondence:

Mio Kobayashi Frisk, MD

Center for Sleep and Vigilance Disorders

Department of Internal Medicine and Clinical Nutrition

University of Gothenburg

Medicinaregatan 8b Box 421

SE-40530, Gothenburg

Sweden

E-mail: mio.kobayashi.frisk@vgregion.se

# Search queries

## 1. CAB Direct (including CAB Abstracts and Global Health)

| **#** | **Block name** | **Search terms** |
| --- | --- | --- |
| **1** | Sleep duration | **(**  ("sleep duration" OR "sleep deprivation" OR "insufficient sleep")  OR  (("quantity" NEAR/2 "sleep") OR ("quantities" NEAR/2 "sleep") OR ("amount" NEAR/2 "sleep") OR "duration" NEAR/2 "sleep") OR ("length" NEAR/2 "sleep") OR ("time" NEAR/2 "sleep") OR ("period" NEAR/2 "sleep") OR ("hours" NEAR/2 "sleep") OR ("minutes" NEAR/2 "sleep") OR ("span" NEAR/2 "sleep"))  OR  (("duration" NEAR/2 "asleep") OR ("length" NEAR/2 "asleep") OR ("time" NEAR/2 "asleep") OR ("period" NEAR/2 "asleep") OR ("hours" NEAR/2 "asleep") OR ("minutes" NEAR/2 "asleep"))  OR  (("quantity" NEAR/2 "sleeping") OR ("quantities" NEAR/2 "sleeping") OR ("amount" NEAR/2 "sleeping") OR ("duration" NEAR/2 "sleeping") OR ("length" NEAR/2 "sleeping") OR ("time" NEAR/2 "sleeping") OR ("period" NEAR/2 "sleeping") OR ("hours" NEAR/2 "sleeping") OR ("minutes" NEAR/2 "sleeping") OR ("span" NEAR/2 "sleeping"))  OR  (("short" NEAR/2 "sleep") OR ("long" NEAR/2 "sleep") OR ("extended" NEAR/2 "sleep"))  OR  (("short" NEAR/2 "sleeper") OR ("long" NEAR/2 "sleeper") OR ("extended" NEAR/2 "sleeper"))  OR  (("short" NEAR/2 "sleepers") OR ("long" NEAR/2 "sleepers") OR ("extended" NEAR/2 "sleepers"))  **)** |
| **2** | Sleep components | **(**  ("sleep" OR "sleep*" OR "wake*" OR "waking" OR "awake")  OR  ("actigraph*" OR "actimetr*" OR "acceleromet*" OR "polysomnograph*" OR "EEG" OR "electroencephalogram" OR "MSLT" OR "MWT" OR "fitbit" OR "dreem" OR "Oura ring" OR "Gen3" OR "Fitbit" OR "Mi band")  OR  ("circadian rhythm" OR "circadian" OR "chronotype*" OR "chronotherap*" OR "eveningness" OR "morningness" OR "evening type*" OR "morning type*" OR "bedtime*" OR "time to bed" OR "time in bed" OR "shuteye" OR "shut-eye" OR "lights off" OR "lights on" OR "shift workers" OR "shift work*" OR "shiftwork*" OR ("shift" NEAR/2 "schedule") OR ("shift" NEAR/2 "schedules") OR ("shift" NEAR/2 "scheduling") OR ("shifting" NEAR/2 "schedule") OR ("shifting" NEAR/2 "schedules") OR ("shifting" NEAR/2 "scheduling") OR ("working" NEAR/2 "hours") OR ("work" NEAR/2 "hours") OR ("work" NEAR/2 "schedule") OR ("work" NEAR/2 "schedules") OR ("work" NEAR/2 "scheduling") OR ("working" NEAR/2 "schedule") OR ("working" NEAR/2 "schedules") OR "jetlag" OR "jet-lag" OR "light*" OR "nois*" OR "WASO" OR "TIB" OR "SE")  OR  ("fatigue*" OR "tired*" OR "somnolence" OR "nap" OR "napping" OR "alert*" OR "ESS" OR "KSS" OR "EDS" OR "day" OR "daytime" OR "night*" OR "drows*" OR "siesta")  OR  ("sleep disorders" OR "insomnia" OR "restless legs syndrome" OR "restless leg syndrome" OR "Willis-Ekbom" OR "Wittmaack-Ekbom" OR "RLS" OR "periodic leg movement*" OR "periodic limb movement*" OR "snoring" OR "snoring" OR "snore" OR "hypersomnia" OR "dyssomnia" OR "parasomnia" OR "narcolepsy" OR "night terror" OR "nightmare*" OR "apnea" OR "apnoea" OR "hypopnea" OR "hypopnea" OR "OSA" OR "OSAHS" OR "AHI" OR "CSA" OR "UARS" OR "upper airway resistance syndrome")  **)** |
| **3** | Multidimensionality | **(**  ("sleep" OR "sleep*")  AND  (  ("RU-SATED" OR "RU_SATED" OR "PSQI")  OR  ("score*" OR "index" OR "indices" OR "multidimensional" OR "multi-dimensional" OR "multi*" OR "dimension*" OR "component*" OR "parameter*" OR "metric*" OR "composite" OR "combination*")  )  **)** |
| **4** | Multidimensional sleep health | **(#1** AND **(#2** OR **#3))** |
| **5** | All-cause outcomes | **(**"mortality" OR "mortality" OR "death*" OR "lethal"**)** |
| **6** | Specific outcomes of interest | **(**  ("SCD" OR "cardiopulmonary arrest" OR "cardiac arrest" OR "heart arrest" OR "asystole" OR "cardiac event*" OR "myocardial infarction" OR "myocardial infarct*" OR "myocardial ischemia" OR "myocardial ischemia" OR "unstable angina" OR "acute coronary syndrome" OR "ACS" OR "AMI" OR "MI" OR "heart failure" OR "heart failure" OR "cardiac failure" OR "myocardial failure" OR "heart decompensation" OR "ventricular dysfunction" OR "CHF")  OR  ("stroke" OR "stroke" OR "cerebral infarct*" OR "cerebrovascular accident*" OR "CVA" OR "brain vascular accident*" OR "cerebrovascular apoplexy" OR "brain ischemia" OR "intracranial hemorrhage" OR "intracranial haemorrhage" OR "cerebral hemorrhage" OR "cerebral haemorrhage")  OR  ("MACE" OR "major adverse cardiovascular event*" OR "infarct*")  **)** |
| **7** | Outcomes | **(#5** OR **#6)** |
| **8** | Full query | **#4** AND **#7**  (( ("sleep deprivation" OR "insufficient sleep") OR (("quantity" NEAR/2 "sleep") OR ("quantities" NEAR/2 "sleep") OR ("amount" NEAR/2 "sleep") OR "duration" NEAR/2 "sleep") OR ("length" NEAR/2 "sleep") OR ("time" NEAR/2 "sleep") OR ("period" NEAR/2 "sleep") OR ("hours" NEAR/2 "sleep") OR ("minutes" NEAR/2 "sleep") OR ("span" NEAR/2 "sleep")) OR (("duration" NEAR/2 "asleep") OR ("length" NEAR/2 "asleep") OR ("time" NEAR/2 "asleep") OR ("period" NEAR/2 "asleep") OR ("hours" NEAR/2 "asleep") OR ("minutes" NEAR/2 "asleep")) OR (("quantity" NEAR/2 "sleeping") OR ("quantities" NEAR/2 "sleeping") OR ("amount" NEAR/2 "sleeping") OR ("duration" NEAR/2 "sleeping") OR ("length" NEAR/2 "sleeping") OR ("time" NEAR/2 "sleeping") OR ("period" NEAR/2 "sleeping") OR ("hours" NEAR/2 "sleeping") OR ("minutes" NEAR/2 "sleeping") OR ("span" NEAR/2 "sleeping")) OR (("short" NEAR/2 "sleep") OR ("long" NEAR/2 "sleep") OR ("extended" NEAR/2 "sleep")) OR (("short" NEAR/2 "sleeper") OR ("long" NEAR/2 "sleeper") OR ("extended" NEAR/2 "sleeper")) OR (("short" NEAR/2 "sleepers") OR ("long" NEAR/2 "sleepers") OR ("extended" NEAR/2 "sleepers")) ) AND (( ("sleep" OR "sleep*" OR "wake*" OR "waking" OR "awake") OR ("actigraph*" OR "actimetr*" OR "acceleromet*" OR "polysomnograph*" OR "EEG" OR "electroencephalogram" OR "MSLT" OR "MWT" OR "fitbit" OR "dreem" OR "Oura ring" OR "Gen3" OR "Fitbit" OR "Mi band") OR ("circadian rhythm" OR "circadian" OR "chronotype*" OR "chronotherap*" OR "eveningness" OR "morningness" OR "evening type*" OR "morning type*" OR "bedtime*" OR "time to bed" OR "time in bed" OR "shuteye" OR "shut-eye" OR "lights off" OR "lights on" OR "shift workers" OR "shift work*" OR "shiftwork*" OR ("shift" NEAR/2 "schedule") OR ("shift" NEAR/2 "schedules") OR ("shift" NEAR/2 "scheduling") OR ("shifting" NEAR/2 "schedule") OR ("shifting" NEAR/2 "schedules") OR ("shifting" NEAR/2 "scheduling") OR ("working" NEAR/2 "hours") OR ("work" NEAR/2 "hours") OR ("work" NEAR/2 "schedule") OR ("work" NEAR/2 "schedules") OR ("work" NEAR/2 "scheduling") OR ("working" NEAR/2 "schedule") OR ("working" NEAR/2 "schedules") OR "jetlag" OR "jet-lag" OR "light*" OR "nois*" OR "WASO" OR "TIB" OR "SE") OR ("fatigue*" OR "tired*" OR "somnolence" OR "nap" OR "napping" OR "alert*" OR "ESS" OR "KSS" OR "EDS" OR "day" OR "daytime" OR "night*" OR "drows*" OR "siesta") OR ("sleep disorders" OR "insomnia" OR "restless legs syndrome" OR "restless leg syndrome" OR "Willis-Ekbom" OR "Wittmaack-Ekbom" OR "RLS" OR "periodic leg movement*" OR "periodic limb movement*" OR "snoring" OR "snoring" OR "snore" OR "hypersomnia" OR "dyssomnia" OR "parasomnia" OR "narcolepsy" OR "night terror" OR "nightmare*" OR "apnea" OR "apnoea" OR "hypopnea" OR "hypopnea" OR "OSA" OR "OSAHS" OR "AHI" OR "CSA" OR "UARS" OR "upper airway resistance syndrome") ) OR ( ("sleep" OR "sleep*") AND ( ("RU-SATED" OR "RU_SATED" OR "PSQI") OR ("score*" OR "index" OR "indices" OR "multidimensional" OR "multi-dimensional" OR "multi*" OR "dimension*" OR "component*" OR "parameter*" OR "metric*" OR "composite" OR "combination*") ) ) )) AND (("mortality" OR "mortality" OR "death*" OR "lethal") OR ( ("SCD" OR "cardiopulmonary arrest" OR "cardiac arrest" OR "heart arrest" OR "asystole" OR "cardiac event*" OR "myocardial infarction" OR "myocardial infarct*" OR "myocardial ischemia" OR "myocardial ischemia" OR "unstable angina" OR "acute coronary syndrome" OR "ACS" OR "AMI" OR "MI" OR "heart failure" OR "heart failure" OR "cardiac failure" OR "myocardial failure" OR "heart decompensation" OR "ventricular dysfunction" OR "CHF") OR ("stroke" OR "stroke" OR "cerebral infarct*" OR "cerebrovascular accident*" OR "CVA" OR "brain vascular accident*" OR "cerebrovascular apoplexy" OR "brain ischemia" OR "intracranial hemorrhage" OR "intracranial haemorrhage" OR "cerebral hemorrhage" OR "cerebral haemorrhage") OR ("MACE" OR "major adverse cardiovascular event*" OR "infarct*") ) ) |

**Abbreviations.** NEAR/2: denotes combinations of two words which are searched to be in any order in relation to each other with up to two words in-between. **Clarifications.** 1) a controlled vocabulary exists for CAB Direct, but it is searched through the same syntax as ordinary search terms; 2) **bold font-weight** visually indicates blocks of search terms or parentheses encapsulating these; 3) **green color** denotes blocks of search terms; 4) dark blue color denotes search terms with the [tiab] field setting without the proximity parameter; 5) light blue color denotes search terms with the [tiab] field setting with the proximity parameter; 6) purple color denotes controlled vocabulary search terms.

## 2. CINAHL

| **#** | **Block name** | **Search terms** |
| --- | --- | --- |
| **1** | Sleep duration | **(**  ((MH "Sleep Duration") OR (MH "Sleep Deprivation") OR TI("sleep deprivation" OR "insufficient sleep") OR AB("sleep deprivation" OR "insufficient sleep"))  OR  TI(("quantity" N2 "sleep") OR ("quantities" N2 "sleep") OR ("amount" N2 "sleep") OR ("duration" N2 "sleep") OR ("length" N2 "sleep") OR ("time" N2 "sleep") OR ("period" N2 "sleep") OR ("hours" N2 "sleep") OR ("minutes" N2 "sleep") OR ("span" N2 "sleep")) OR AB(("quantity" N2 "sleep") OR ("quantities" N2 "sleep") OR ("amount" N2 "sleep") OR ("duration" N2 "sleep") OR ("length" N2 "sleep") OR ("time" N2 "sleep") OR ("period" N2 "sleep") OR ("hours" N2 "sleep") OR ("minutes" N2 "sleep") OR ("span" N2 "sleep"))  OR  TI(("duration" N2 "asleep") OR ("length" N2 "asleep") OR ("time" N2 "asleep") OR ("period" N2 "asleep") OR ("hours" N2 "asleep") OR ("minutes asleep")) OR AB(("duration" N2 "asleep") OR ("length" N2 "asleep") OR ("time" N2 "asleep") OR ("period" N2 "asleep") OR ("hours" N2 "asleep") OR ("minutes asleep"))  OR  TI(("quantity" N2 "sleeping") OR ("quantities" N2 "sleeping") OR ("amount" N2 "sleeping") OR ("duration" N2 "sleeping") OR ("length" N2 "sleeping") OR ("time" N2 "sleeping") OR ("period" N2 "sleeping") OR ("hours" N2 "sleeping") OR ("minutes" N2 "sleeping") OR ("span" N2 "sleeping")) OR AB(("quantity" N2 "sleeping") OR ("quantities" N2 "sleeping") OR ("amount" N2 "sleeping") OR ("duration" N2 "sleeping") OR ("length" N2 "sleeping") OR ("time" N2 "sleeping") OR ("period" N2 "sleeping") OR ("hours" N2 "sleeping") OR ("minutes" N2 "sleeping") OR ("span" N2 "sleeping"))  OR  TI(("short" N2 "sleep") OR ("long" N2 "sleep") OR ("extended" N2 "sleep")) OR AB(("short" N2 "sleep") OR ("long" N2 "sleep") OR ("extended" N2 "sleep"))  OR  TI(("short" N2 "sleeper") OR ("long" N2 "sleeper") OR ("extended" N2 "sleeper")) OR AB(("short" N2 "sleeper") OR ("long" N2 "sleeper") OR ("extended" N2 "sleeper"))  OR  TI(("short" N2 "sleepers") OR ("long" N2 "sleepers") OR ("extended" N2 "sleepers")) OR AB(("short" N2 "sleepers") OR ("long" N2 "sleepers") OR ("extended" N2 "sleepers"))  **)** |
| **2** | Sleep components | **(**  ((MH "Sleep+") OR TI("sleep*" OR "wake*" OR "waking" OR "awake") OR AB("sleep*" OR "wake*" OR "waking" OR "awake"))  OR  ((MH "Polysomnography") OR (MH "Actigraphy") OR TI("actigraph*" OR "actimetr*" OR "acceleromet*" OR "polysomnograph*" OR "EEG" OR "electroencephalogram" OR "MSLT" OR "MWT" OR "fitbit" OR "dreem" OR "Oura ring" OR "Gen3" OR "Fitbit" OR "Mi band") OR AB("actigraph*" OR "actimetr*" OR "acceleromet*" OR "polysomnograph*" OR "EEG" OR "electroencephalogram" OR "MSLT" OR "MWT" OR "fitbit" OR "dreem" OR "Oura ring" OR "Gen3" OR "Fitbit" OR "Mi band"))  OR  ((MH "Circadian Rhythm+") OR (MH "Shiftwork") OR TI("circadian" OR "chronotype*" OR "chronotherap*" OR "eveningness" OR "morningness" OR "evening type*" OR "morning type*" OR "bedtime*" OR "time to bed" OR "time in bed" OR "shuteye" OR "shut-eye" OR "lights off" OR "lights on" OR "shift work*" OR "shiftwork*" OR ("shift" N2 "schedule") OR ("shift" N2 "schedules") OR ("shift" N2 "scheduling") OR ("shifting" N2 "schedule") OR ("shifting" N2 "schedules") OR ("shifting" N2 "scheduling") OR ("working" N2 "hours") OR ("work" N2 "hours") OR ("work" N2 "schedule") OR ("work" N2 "schedules") OR ("work" N2 "scheduling") OR ("working" N2 "schedule") OR ("working" N2 "schedules") OR "jetlag" OR "jet-lag" OR "light*" OR "nois*" OR "WASO" OR "TIB" OR "SE") OR AB("circadian" OR "chronotype*" OR "chronotherap*" OR "eveningness" OR "morningness" OR "evening type*" OR "morning type*" OR "bedtime*" OR "time to bed" OR "time in bed" OR "shuteye" OR "shut-eye" OR "lights off" OR "lights on" OR "shift work*" OR "shiftwork*" OR ("shift" N2 "schedule") OR ("shift" N2 "schedules") OR ("shift" N2 "scheduling") OR ("shifting" N2 "schedule") OR ("shifting" N2 "schedules") OR ("shifting" N2 "scheduling") OR ("working" N2 "hours") OR ("work" N2 "hours") OR ("work" N2 "schedule") OR ("work" N2 "schedules") OR ("work" N2 "scheduling") OR ("working" N2 "schedule") OR ("working" N2 "schedules") OR "jetlag" OR "jet-lag" OR "light*" OR "nois*" OR "WASO" OR "TIB" OR "SE"))  OR  ((MH "Sleepiness") OR TI("fatigue*" OR "tired*" OR "somnolence" OR "nap" OR "napping" OR "alert*" OR "ESS" OR "KSS" OR "EDS" OR "day" OR "daytime" OR "night*" OR "drows*" OR "siesta") OR AB("fatigue*" OR "tired*" OR "somnolence" OR "nap" OR "napping" OR "alert*" OR "ESS" OR "KSS" OR "EDS" OR "day" OR "daytime" OR "night*" OR "drows*" OR "siesta"))  OR  ((MH "Sleep Disorders+") OR (MH "Apnea+") OR (MH "Snoring") OR TI("insomnia" OR "restless legs syndrome" OR "restless leg syndrome" OR "Willis-Ekbom" OR "Wittmaack-Ekbom" OR "RLS" OR "periodic leg movement*" OR "periodic limb movement*" OR "snoring" OR "snore" OR "hypersomnia" OR "dyssomnia" OR "parasomnia" OR "narcolepsy" OR "night terror" OR "nightmare*" OR "apnea" OR "apnoea" OR "hypopnea" OR "hypopnea" OR "OSA" OR "OSAHS" OR "AHI" OR "CSA" OR "UARS" OR "upper airway resistance syndrome") OR AB("insomnia" OR "restless legs syndrome" OR "restless leg syndrome" OR "Willis-Ekbom" OR "Wittmaack-Ekbom" OR "RLS" OR "periodic leg movement*" OR "periodic limb movement*" OR "snoring" OR "snore" OR "hypersomnia" OR "dyssomnia" OR "parasomnia" OR "narcolepsy" OR "night terror" OR "nightmare*" OR "apnea" OR "apnoea" OR "hypopnea" OR "hypopnea" OR "OSA" OR "OSAHS" OR "AHI" OR "CSA" OR "UARS" OR "upper airway resistance syndrome"))  **)** |
| **3** | Multidimensionality | **(**  ((MH "Sleep+") OR TI("sleep*") OR AB("sleep*"))  AND  (  TI("RU-SATED" OR "RU_SATED" OR "PSQI") OR AB("RU-SATED" OR "RU_SATED" OR "PSQI")  OR  TI("score*" OR "index" OR "indices" OR "multidimensional" OR "multi-dimensional" OR "multi*" OR "dimension*" OR "component*" OR "parameter*" OR "metric*" OR "composite" OR "combination*") OR AB("score*" OR "index" OR "indices" OR "multidimensional" OR "multi-dimensional" OR "multi*" OR "dimension*" OR "component*" OR "parameter*" OR "metric*" OR "composite" OR "combination*")  )  **)** |
| **4** | Multidimensional sleep health | **(#1** AND **(#2** OR **#3))** |
| **5** | All-cause outcomes | **(**(MH "Mortality+") OR TI("mortality" OR "death*" OR "lethal") OR AB("mortality" OR "death*" OR "lethal")**)** |
| **6** | Specific outcomes of interest | **(**  ((MH "Heart Arrest+") OR (MH "Myocardial Infarction+") OR (MH "Myocardial Ischemia+") OR (MH "Heart Failure+") OR TI("SCD" OR "cardiopulmonary arrest" OR "cardiac arrest" OR "heart arrest" OR "asystole" OR "cardiac event*" OR "myocardial infarct*" OR "myocardial ischemia" OR "unstable angina" OR "acute coronary syndrome" OR "ACS" OR "AMI" OR "MI" OR "heart failure" OR "cardiac failure" OR "myocardial failure" OR "heart decompensation" OR "ventricular dysfunction" OR "CHF") OR AB("SCD" OR "cardiopulmonary arrest" OR "cardiac arrest" OR "heart arrest" OR "asystole" OR "cardiac event*" OR "myocardial infarct*" OR "myocardial ischemia" OR "unstable angina" OR "acute coronary syndrome" OR "ACS" OR "AMI" OR "MI" OR "heart failure" OR "cardiac failure" OR "myocardial failure" OR "heart decompensation" OR "ventricular dysfunction" OR "CHF"))  OR  ((MH "Stroke+") OR TI("stroke" OR "cerebral infarct*" OR "cerebrovascular accident*" OR "CVA" OR "brain vascular accident*" OR "cerebrovascular apoplexy" OR "brain ischemia" OR "intracranial hemorrhage" OR "intracranial haemorrhage" OR "cerebral hemorrhage" OR "cerebral haemorrhage") OR AB("stroke" OR "cerebral infarct*" OR "cerebrovascular accident*" OR "CVA" OR "brain vascular accident*" OR "cerebrovascular apoplexy" OR "brain ischemia" OR "intracranial hemorrhage" OR "intracranial haemorrhage" OR "cerebral hemorrhage" OR "cerebral haemorrhage"))  OR  TI("MACE" OR "major adverse cardiovascular event*" OR "infarct*") OR AB("MACE" OR "major adverse cardiovascular event*" OR "infarct*")  **)** |
| **7** | Outcomes | **(#5** OR **#6)** |
| **8** | Full query | **#4** AND **#7**  (( ((MH "Sleep Duration") OR (MH "Sleep Deprivation") OR TI("sleep deprivation" OR "insufficient sleep") OR AB("sleep deprivation" OR "insufficient sleep")) OR TI(("quantity" N2 "sleep") OR ("quantities" N2 "sleep") OR ("amount" N2 "sleep") OR ("duration" N2 "sleep") OR ("length" N2 "sleep") OR ("time" N2 "sleep") OR ("period" N2 "sleep") OR ("hours" N2 "sleep") OR ("minutes" N2 "sleep") OR ("span" N2 "sleep")) OR AB(("quantity" N2 "sleep") OR ("quantities" N2 "sleep") OR ("amount" N2 "sleep") OR ("duration" N2 "sleep") OR ("length" N2 "sleep") OR ("time" N2 "sleep") OR ("period" N2 "sleep") OR ("hours" N2 "sleep") OR ("minutes" N2 "sleep") OR ("span" N2 "sleep")) OR TI(("duration" N2 "asleep") OR ("length" N2 "asleep") OR ("time" N2 "asleep") OR ("period" N2 "asleep") OR ("hours" N2 "asleep") OR ("minutes asleep")) OR AB(("duration" N2 "asleep") OR ("length" N2 "asleep") OR ("time" N2 "asleep") OR ("period" N2 "asleep") OR ("hours" N2 "asleep") OR ("minutes asleep")) OR TI(("quantity" N2 "sleeping") OR ("quantities" N2 "sleeping") OR ("amount" N2 "sleeping") OR ("duration" N2 "sleeping") OR ("length" N2 "sleeping") OR ("time" N2 "sleeping") OR ("period" N2 "sleeping") OR ("hours" N2 "sleeping") OR ("minutes" N2 "sleeping") OR ("span" N2 "sleeping")) OR AB(("quantity" N2 "sleeping") OR ("quantities" N2 "sleeping") OR ("amount" N2 "sleeping") OR ("duration" N2 "sleeping") OR ("length" N2 "sleeping") OR ("time" N2 "sleeping") OR ("period" N2 "sleeping") OR ("hours" N2 "sleeping") OR ("minutes" N2 "sleeping") OR ("span" N2 "sleeping")) OR TI(("short" N2 "sleep") OR ("long" N2 "sleep") OR ("extended" N2 "sleep")) OR AB(("short" N2 "sleep") OR ("long" N2 "sleep") OR ("extended" N2 "sleep")) OR TI(("short" N2 "sleeper") OR ("long" N2 "sleeper") OR ("extended" N2 "sleeper")) OR AB(("short" N2 "sleeper") OR ("long" N2 "sleeper") OR ("extended" N2 "sleeper")) OR TI(("short" N2 "sleepers") OR ("long" N2 "sleepers") OR ("extended" N2 "sleepers")) OR AB(("short" N2 "sleepers") OR ("long" N2 "sleepers") OR ("extended" N2 "sleepers")) ) AND (( ((MH "Sleep+") OR TI("sleep*" OR "wake*" OR "waking" OR "awake") OR AB("sleep*" OR "wake*" OR "waking" OR "awake")) OR ((MH "Polysomnography") OR (MH "Actigraphy") OR TI("actigraph*" OR "actimetr*" OR "acceleromet*" OR "polysomnograph*" OR "EEG" OR "electroencephalogram" OR "MSLT" OR "MWT" OR "fitbit" OR "dreem" OR "Oura ring" OR "Gen3" OR "Fitbit" OR "Mi band") OR AB("actigraph*" OR "actimetr*" OR "acceleromet*" OR "polysomnograph*" OR "EEG" OR "electroencephalogram" OR "MSLT" OR "MWT" OR "fitbit" OR "dreem" OR "Oura ring" OR "Gen3" OR "Fitbit" OR "Mi band")) OR ((MH "Circadian Rhythm+") OR (MH "Shiftwork") OR TI("circadian" OR "chronotype*" OR "chronotherap*" OR "eveningness" OR "morningness" OR "evening type*" OR "morning type*" OR "bedtime*" OR "time to bed" OR "time in bed" OR "shuteye" OR "shut-eye" OR "lights off" OR "lights on" OR "shift work*" OR "shiftwork*" OR ("shift" N2 "schedule") OR ("shift" N2 "schedules") OR ("shift" N2 "scheduling") OR ("shifting" N2 "schedule") OR ("shifting" N2 "schedules") OR ("shifting" N2 "scheduling") OR ("working" N2 "hours") OR ("work" N2 "hours") OR ("work" N2 "schedule") OR ("work" N2 "schedules") OR ("work" N2 "scheduling") OR ("working" N2 "schedule") OR ("working" N2 "schedules") OR "jetlag" OR "jet-lag" OR "light*" OR "nois*" OR "WASO" OR "TIB" OR "SE") OR AB("circadian" OR "chronotype*" OR "chronotherap*" OR "eveningness" OR "morningness" OR "evening type*" OR "morning type*" OR "bedtime*" OR "time to bed" OR "time in bed" OR "shuteye" OR "shut-eye" OR "lights off" OR "lights on" OR "shift work*" OR "shiftwork*" OR ("shift" N2 "schedule") OR ("shift" N2 "schedules") OR ("shift" N2 "scheduling") OR ("shifting" N2 "schedule") OR ("shifting" N2 "schedules") OR ("shifting" N2 "scheduling") OR ("working" N2 "hours") OR ("work" N2 "hours") OR ("work" N2 "schedule") OR ("work" N2 "schedules") OR ("work" N2 "scheduling") OR ("working" N2 "schedule") OR ("working" N2 "schedules") OR "jetlag" OR "jet-lag" OR "light*" OR "nois*" OR "WASO" OR "TIB" OR "SE")) OR ((MH "Sleepiness") OR TI("fatigue*" OR "tired*" OR "somnolence" OR "nap" OR "napping" OR "alert*" OR "ESS" OR "KSS" OR "EDS" OR "day" OR "daytime" OR "night*" OR "drows*" OR "siesta") OR AB("fatigue*" OR "tired*" OR "somnolence" OR "nap" OR "napping" OR "alert*" OR "ESS" OR "KSS" OR "EDS" OR "day" OR "daytime" OR "night*" OR "drows*" OR "siesta")) OR ((MH "Sleep Disorders+") OR (MH "Apnea+") OR (MH "Snoring") OR TI("insomnia" OR "restless legs syndrome" OR "restless leg syndrome" OR "Willis-Ekbom" OR "Wittmaack-Ekbom" OR "RLS" OR "periodic leg movement*" OR "periodic limb movement*" OR "snoring" OR "snore" OR "hypersomnia" OR "dyssomnia" OR "parasomnia" OR "narcolepsy" OR "night terror" OR "nightmare*" OR "apnea" OR "apnoea" OR "hypopnea" OR "hypopnea" OR "OSA" OR "OSAHS" OR "AHI" OR "CSA" OR "UARS" OR "upper airway resistance syndrome") OR AB("insomnia" OR "restless legs syndrome" OR "restless leg syndrome" OR "Willis-Ekbom" OR "Wittmaack-Ekbom" OR "RLS" OR "periodic leg movement*" OR "periodic limb movement*" OR "snoring" OR "snore" OR "hypersomnia" OR "dyssomnia" OR "parasomnia" OR "narcolepsy" OR "night terror" OR "nightmare*" OR "apnea" OR "apnoea" OR "hypopnea" OR "hypopnea" OR "OSA" OR "OSAHS" OR "AHI" OR "CSA" OR "UARS" OR "upper airway resistance syndrome")) ) OR ( ((MH "Sleep+") OR TI("sleep*") OR AB("sleep*")) AND ( TI("RU-SATED" OR "RU_SATED" OR "PSQI") OR AB("RU-SATED" OR "RU_SATED" OR "PSQI") OR TI("score*" OR "index" OR "indices" OR "multidimensional" OR "multi-dimensional" OR "multi*" OR "dimension*" OR "component*" OR "parameter*" OR "metric*" OR "composite" OR "combination*") OR AB("score*" OR "index" OR "indices" OR "multidimensional" OR "multi-dimensional" OR "multi*" OR "dimension*" OR "component*" OR "parameter*" OR "metric*" OR "composite" OR "combination*") ) ))) AND (((MH "Mortality+") OR TI("mortality" OR "death*" OR "lethal") OR AB("mortality" OR "death*" OR "lethal")) OR ( ((MH "Heart Arrest+") OR (MH "Myocardial Infarction+") OR (MH "Myocardial Ischemia+") OR (MH "Heart Failure+") OR TI("SCD" OR "cardiopulmonary arrest" OR "cardiac arrest" OR "heart arrest" OR "asystole" OR "cardiac event*" OR "myocardial infarct*" OR "myocardial ischemia" OR "unstable angina" OR "acute coronary syndrome" OR "ACS" OR "AMI" OR "MI" OR "heart failure" OR "cardiac failure" OR "myocardial failure" OR "heart decompensation" OR "ventricular dysfunction" OR "CHF") OR AB("SCD" OR "cardiopulmonary arrest" OR "cardiac arrest" OR "heart arrest" OR "asystole" OR "cardiac event*" OR "myocardial infarct*" OR "myocardial ischemia" OR "unstable angina" OR "acute coronary syndrome" OR "ACS" OR "AMI" OR "MI" OR "heart failure" OR "cardiac failure" OR "myocardial failure" OR "heart decompensation" OR "ventricular dysfunction" OR "CHF")) OR ((MH "Stroke+") OR TI("stroke" OR "cerebral infarct*" OR "cerebrovascular accident*" OR "CVA" OR "brain vascular accident*" OR "cerebrovascular apoplexy" OR "brain ischemia" OR "intracranial hemorrhage" OR "intracranial haemorrhage" OR "cerebral hemorrhage" OR "cerebral haemorrhage") OR AB("stroke" OR "cerebral infarct*" OR "cerebrovascular accident*" OR "CVA" OR "brain vascular accident*" OR "cerebrovascular apoplexy" OR "brain ischemia" OR "intracranial hemorrhage" OR "intracranial haemorrhage" OR "cerebral hemorrhage" OR "cerebral haemorrhage")) OR TI("MACE" OR "major adverse cardiovascular event*" OR "infarct*") OR AB("MACE" OR "major adverse cardiovascular event*" OR "infarct*") )) |

**Abbreviations.** AB: search to be made in the abstract field. MH: search to be made by CINAHL Subject Headings, a controlled vocabulary (search terms with "+" at the end include underlying subject headings). N2: search with a proximity parameter, which matches the word before and after "N2" in any order, with up to two words in-between. TI: search to be made in the title field. **Clarifications.** 1) **bold font-weight** visually indicates blocks of search terms or parentheses encapsulating these; 2) **green color** denotes blocks of search terms; 3) dark blue color denotes search terms with the AB or TI field setting without the proximity parameter; 4) light blue color denotes search terms with the AB or TI field setting with the proximity parameter; 5) purple color denotes controlled vocabulary search terms.

## 3. Embase

| **#** | **Block name** | **Search terms** |
| --- | --- | --- |
| **1** | Sleep duration | **(**  ('sleep time'/exp OR 'sleep deprivation'/exp OR ("sleep deprivation" OR "insufficient sleep"):ti,ab)  OR  (("quantity" NEAR/2 "sleep") OR ("quantities" NEAR/2 "sleep") OR ("amount" NEAR/2 "sleep") OR ("duration" NEAR/2 "sleep") OR ("length" NEAR/2 "sleep") OR ("time" NEAR/2 "sleep") OR ("period" NEAR/2 "sleep") OR ("hours" NEAR/2 "sleep") OR ("minutes" NEAR/2 "sleep") OR ("span" NEAR/2 "sleep")):ti,ab  OR  (("duration" NEAR/2 "asleep") OR ("length" NEAR/2 "asleep") OR ("time" NEAR/2 "asleep") OR ("period" NEAR/2 "asleep") OR ("hours" NEAR/2 "asleep") OR ("minutes asleep")):ti,ab  OR  (("quantity" NEAR/2 "sleeping") OR ("quantities" NEAR/2 "sleeping") OR ("amount" NEAR/2 "sleeping") OR ("duration" NEAR/2 "sleeping") OR ("length" NEAR/2 "sleeping") OR ("time" NEAR/2 "sleeping") OR ("period" NEAR/2 "sleeping") OR ("hours" NEAR/2 "sleeping") OR ("minutes" NEAR/2 "sleeping") OR ("span" NEAR/2 "sleeping")):ti,ab  OR  (("short" NEAR/2 "sleep") OR ("long" NEAR/2 "sleep") OR ("extended" NEAR/2 "sleep")):ti,ab  OR  (("short" NEAR/2 "sleeper") OR ("long" NEAR/2 "sleeper") OR ("extended" NEAR/2 "sleeper")):ti,ab  OR  (("short" NEAR/2 "sleepers") OR ("long" NEAR/2 "sleepers") OR ("extended" NEAR/2 "sleepers")):ti,ab  **)** |
| **2** | Sleep components | **(**  ('sleep'/exp OR ("sleep*" OR "wake*" OR "waking" OR "awake"):ti,ab)  OR  ('polysomnography'/exp OR 'actimetry'/exp OR ("actigraph*" OR "actimetr*" OR "acceleromet*" OR "polysomnograph*" OR "EEG" OR "electroencephalogram" OR "MSLT" OR "MWT" OR "fitbit" OR "dreem" OR "Oura ring" OR "Gen3" OR "Fitbit" OR "Mi band"):ti,ab)  OR  ('circadian rhythm'/exp OR 'shift work'/exp OR ("circadian" OR "chronotype*" OR "chronotherap*" OR "eveningness" OR "morningness" OR "evening type*" OR "morning type*" OR "bedtime*" OR "time to bed" OR "time in bed" OR "shuteye" OR "shut-eye" OR "lights off" OR "lights on" OR "shift work*" OR "shiftwork*" OR ("shift" NEAR/2 "schedule") OR ("shift" NEAR/2 "schedules") OR ("shift" NEAR/2 "scheduling") OR ("shifting" NEAR/2 "schedule") OR ("shifting" NEAR/2 "schedules") OR ("shifting" NEAR/2 "scheduling") OR ("working" NEAR/2 "hours") OR ("work" NEAR/2 "hours") OR ("work" NEAR/2 "schedule") OR ("work" NEAR/2 "schedules") OR ("work" NEAR/2 "scheduling") OR ("working" NEAR/2 "schedule") OR ("working" NEAR/2 "schedules") OR "jetlag" OR "jet-lag" OR "light*" OR "nois*" OR "WASO" OR "TIB" OR "SE"):ti,ab)  OR  ('somnolence'/exp OR ("fatigue*" OR "tired*" OR "somnolence" OR "nap" OR "napping" OR "alert*" OR "ESS" OR "KSS" OR "EDS" OR "day" OR "daytime" OR "night*" OR "drows*" OR "siesta"):ti,ab)  OR  ('sleep disorder'/exp OR 'apnea'/exp OR 'snoring'/exp OR ("insomnia" OR "restless legs syndrome" OR "restless leg syndrome" OR "Willis-Ekbom" OR "Wittmaack-Ekbom" OR "RLS" OR "periodic leg movement*" OR "periodic limb movement*" OR "snoring" OR "snore" OR "hypersomnia" OR "dyssomnia" OR "parasomnia" OR "narcolepsy" OR "night terror" OR "nightmare*" OR "apnea" OR "apnoea" OR "hypopnea" OR "hypopnea" OR "OSA" OR "OSAHS" OR "AHI" OR "CSA" OR "UARS" OR "upper airway resistance syndrome"):ti,ab)  **)** |
| **3** | Multidimensionality | **(**  ('sleep'/exp OR ("sleep*"):ti,ab)  AND  (  ("RU-SATED" OR "RU_SATED" OR "PSQI"):ti,ab  OR  ("score*" OR "index" OR "indices" OR "multidimensional" OR "multi-dimensional" OR "multi*" OR "dimension*" OR "component*" OR "parameter*" OR "metric*" OR "composite" OR "combination*"):ti,ab  )  **)** |
| **4** | Multidimensional sleep health | **(#1** AND **(#2** OR **#3))** |
| **5** | All-cause outcomes | **(**'mortality'/exp OR ("mortality" OR "death*" OR "lethal"):ti,ab**)** |
| **6** | Specific outcomes of interest | **(**  ('heart arrest'/exp OR 'heart infarction'/exp OR 'heart muscle ischemia'/exp OR 'heart failure'/exp OR ("SCD" OR "cardiopulmonary arrest" OR "cardiac arrest" OR "heart arrest" OR "asystole" OR "cardiac event*" OR "myocardial infarct*" OR "myocardial ischemia" OR "unstable angina" OR "acute coronary syndrome" OR "ACS" OR "AMI" OR "MI" OR "heart failure" OR "cardiac failure" OR "myocardial failure" OR "heart decompensation" OR "ventricular dysfunction" OR "CHF"):ti,ab)  OR  ('cerebrovascular accident'/exp OR ("stroke" OR "cerebral infarct*" OR "cerebrovascular accident*" OR "CVA" OR "brain vascular accident*" OR "cerebrovascular apoplexy" OR "brain ischemia" OR "intracranial hemorrhage" OR "intracranial haemorrhage" OR "cerebral hemorrhage" OR "cerebral haemorrhage"):ti,ab)  OR  ("MACE" OR "major adverse cardiovascular event*" OR "infarct*"):ti,ab  **)** |
| **7** | Outcomes | **(#5** OR **#6)** |
| **8** | Full query | **#4** AND **#7**  (( ('sleep time'/exp OR 'sleep deprivation'/exp OR ("sleep deprivation" OR "insufficient sleep"):ti,ab) OR (("quantity" NEAR/2 "sleep") OR ("quantities" NEAR/2 "sleep") OR ("amount" NEAR/2 "sleep") OR ("duration" NEAR/2 "sleep") OR ("length" NEAR/2 "sleep") OR ("time" NEAR/2 "sleep") OR ("period" NEAR/2 "sleep") OR ("hours" NEAR/2 "sleep") OR ("minutes" NEAR/2 "sleep") OR ("span" NEAR/2 "sleep")):ti,ab OR (("duration" NEAR/2 "asleep") OR ("length" NEAR/2 "asleep") OR ("time" NEAR/2 "asleep") OR ("period" NEAR/2 "asleep") OR ("hours" NEAR/2 "asleep") OR ("minutes asleep")):ti,ab OR (("quantity" NEAR/2 "sleeping") OR ("quantities" NEAR/2 "sleeping") OR ("amount" NEAR/2 "sleeping") OR ("duration" NEAR/2 "sleeping") OR ("length" NEAR/2 "sleeping") OR ("time" NEAR/2 "sleeping") OR ("period" NEAR/2 "sleeping") OR ("hours" NEAR/2 "sleeping") OR ("minutes" NEAR/2 "sleeping") OR ("span" NEAR/2 "sleeping")):ti,ab OR (("short" NEAR/2 "sleep") OR ("long" NEAR/2 "sleep") OR ("extended" NEAR/2 "sleep")):ti,ab OR (("short" NEAR/2 "sleeper") OR ("long" NEAR/2 "sleeper") OR ("extended" NEAR/2 "sleeper")):ti,ab OR (("short" NEAR/2 "sleepers") OR ("long" NEAR/2 "sleepers") OR ("extended" NEAR/2 "sleepers")):ti,ab ) AND (( ('sleep'/exp OR ("sleep*" OR "wake*" OR "waking" OR "awake"):ti,ab) OR ('polysomnography'/exp OR 'actimetry'/exp OR ("actigraph*" OR "actimetr*" OR "acceleromet*" OR "polysomnograph*" OR "EEG" OR "electroencephalogram" OR "MSLT" OR "MWT" OR "fitbit" OR "dreem" OR "Oura ring" OR "Gen3" OR "Fitbit" OR "Mi band"):ti,ab) OR ('circadian rhythm'/exp OR 'shift work'/exp OR ("circadian" OR "chronotype*" OR "chronotherap*" OR "eveningness" OR "morningness" OR "evening type*" OR "morning type*" OR "bedtime*" OR "time to bed" OR "time in bed" OR "shuteye" OR "shut-eye" OR "lights off" OR "lights on" OR "shift work*" OR "shiftwork*" OR ("shift" NEAR/2 "schedule") OR ("shift" NEAR/2 "schedules") OR ("shift" NEAR/2 "scheduling") OR ("shifting" NEAR/2 "schedule") OR ("shifting" NEAR/2 "schedules") OR ("shifting" NEAR/2 "scheduling") OR ("working" NEAR/2 "hours") OR ("work" NEAR/2 "hours") OR ("work" NEAR/2 "schedule") OR ("work" NEAR/2 "schedules") OR ("work" NEAR/2 "scheduling") OR ("working" NEAR/2 "schedule") OR ("working" NEAR/2 "schedules") OR "jetlag" OR "jet-lag" OR "light*" OR "nois*" OR "WASO" OR "TIB" OR "SE"):ti,ab) OR ('somnolence'/exp OR ("fatigue*" OR "tired*" OR "somnolence" OR "nap" OR "napping" OR "alert*" OR "ESS" OR "KSS" OR "EDS" OR "day" OR "daytime" OR "night*" OR "drows*" OR "siesta"):ti,ab) OR ('sleep disorder'/exp OR 'apnea'/exp OR 'snoring'/exp OR ("insomnia" OR "restless legs syndrome" OR "restless leg syndrome" OR "Willis-Ekbom" OR "Wittmaack-Ekbom" OR "RLS" OR "periodic leg movement*" OR "periodic limb movement*" OR "snoring" OR "snore" OR "hypersomnia" OR "dyssomnia" OR "parasomnia" OR "narcolepsy" OR "night terror" OR "nightmare*" OR "apnea" OR "apnoea" OR "hypopnea" OR "hypopnea" OR "OSA" OR "OSAHS" OR "AHI" OR "CSA" OR "UARS" OR "upper airway resistance syndrome"):ti,ab) ) OR ( ('sleep'/exp OR ("sleep*"):ti,ab) AND ( ("RU-SATED" OR "RU_SATED" OR "PSQI"):ti,ab OR ("score*" OR "index" OR "indices" OR "multidimensional" OR "multi-dimensional" OR "multi*" OR "dimension*" OR "component*" OR "parameter*" OR "metric*" OR "composite" OR "combination*"):ti,ab ) ))) AND (('mortality'/exp OR ("mortality" OR "death*" OR "lethal"):ti,ab) OR ( ('heart arrest'/exp OR 'heart infarction'/exp OR 'heart muscle ischemia'/exp OR 'heart failure'/exp OR ("SCD" OR "cardiopulmonary arrest" OR "cardiac arrest" OR "heart arrest" OR "asystole" OR "cardiac event*" OR "myocardial infarct*" OR "myocardial ischemia" OR "unstable angina" OR "acute coronary syndrome" OR "ACS" OR "AMI" OR "MI" OR "heart failure" OR "cardiac failure" OR "myocardial failure" OR "heart decompensation" OR "ventricular dysfunction" OR "CHF"):ti,ab) OR ('cerebrovascular accident'/exp OR ("stroke" OR "cerebral infarct*" OR "cerebrovascular accident*" OR "CVA" OR "brain vascular accident*" OR "cerebrovascular apoplexy" OR "brain ischemia" OR "intracranial hemorrhage" OR "intracranial haemorrhage" OR "cerebral hemorrhage" OR "cerebral haemorrhage"):ti,ab) OR ("MACE" OR "major adverse cardiovascular event*" OR "infarct*"):ti,ab )) |

**Abbreviations.** /exp: expand the controlled vocabulary search to underlying subject headings. NEAR/2: search for word before and after in any order with up to two words in-between (proximity parameter). :ti,ab: search to be made in the title and abstract fields. **Clarifications.** 1) **bold font-weight** visually indicates blocks of search terms or parentheses encapsulating these; 2) **green color** denotes blocks of search terms; 3) dark blue color denotes search terms with the :ti,ab field setting without the proximity parameter; 4) light blue color denotes search terms with the :ti,ab field setting with the proximity parameter; 5) purple color denotes controlled vocabulary search terms.

## 4. Google Scholar

| **Block name** | **Search terms** |
| --- | --- |
| Sleep duration | sleep (deprivation\|duration\|length\|time\|quantity) |
| Sleep components and multi-dimensional sleep health | (timing\|quality\|regularity\|satisfaction\|efficiency\|alertness\|work\|disorder\|snoring\|apnea\|index\|composite\|multidimensional\|score) |
| Outcomes | (mortality\|death\|MACE\|stroke\|infarction\|"acute coronary syndrome"\|"heart failure") |
| Full query | sleep (deprivation\|duration\|length\|time\|quantity) (timing\|quality\|regularity\|satisfaction\|efficiency\|alertness\|work\|disorder\|snoring\|apnea\|index\|composite\|multidimensional\|score) (mortality\|death\|MACE\|stroke\|infarction\|"acute coronary syndrome"\|"heart failure") |

**Abbreviations.** |: pipe operator (equivalent to the "OR" Boolean operator). **Clarifications.** 1) given the 256 character limit of a search query in Google Scholar, only the most essential search terms are defined; 2) no controlled vocabulary exists for Google Scholar; 2) regular space (" ") is equivalent to the "AND" Boolean operator, which is why no such operator is defined in this search query.

## 5. PsycInfo

| **#** |  | **Block name** | **Search terms** |
| --- | --- | --- | --- |
| **1** |  | Sleep duration | **(**  (MAINSUBJECT.EXACT.EXPLODE("Sleep Deprivation") OR TI,AB("sleep deprivation") OR TI,AB("insufficient sleep"))  OR  (TI,AB("quantity" NEAR/2 "sleep") OR TI,AB("quantities" NEAR/2 "sleep") OR TI,AB("amount" NEAR/2 "sleep") OR TI,AB("duration" NEAR/2 "sleep") OR TI,AB("length" NEAR/2 "sleep") OR TI,AB("time" NEAR/2 "sleep") OR TI,AB("period" NEAR/2 "sleep") OR TI,AB("hours" NEAR/2 "sleep") OR TI,AB("minutes" NEAR/2 "sleep") OR TI,AB("span" NEAR/2 "sleep"))  OR  (TI,AB("duration" NEAR/2 "asleep") OR TI,AB("length" NEAR/2 "asleep") OR TI,AB("time" NEAR/2 "asleep") OR TI,AB("period" NEAR/2 "asleep") OR TI,AB("hours" NEAR/2 "asleep") OR TI,AB("minutes" NEAR/2 "asleep"))  OR  (TI,AB("quantity" NEAR/2 "sleeping") OR TI,AB("quantities" NEAR/2 "sleeping") OR TI,AB("amount" NEAR/2 "sleeping") OR TI,AB("duration" NEAR/2 "sleeping") OR TI,AB("length" NEAR/2 "sleeping") OR TI,AB("time" NEAR/2 "sleeping") OR TI,AB("period" NEAR/2 "sleeping") OR TI,AB("hours" NEAR/2 "sleeping") OR TI,AB("minutes" NEAR/2 "sleeping") OR TI,AB("span" NEAR/2 "sleeping"))  OR  (TI,AB("short" NEAR/2 "sleep") OR TI,AB("long" NEAR/2 "sleep") OR TI,AB("extended" NEAR/2 "sleep"))  OR  (TI,AB("short" NEAR/2 "sleeper") OR TI,AB("long" NEAR/2 "sleeper") OR TI,AB("extended" NEAR/2 "sleeper"))  OR  (TI,AB("short" NEAR/2 "sleepers") OR TI,AB("long" NEAR/2 "sleepers") OR TI,AB("extended" NEAR/2 "sleepers"))  **)** |
| **2** |  | Sleep components | **(**  (MAINSUBJECT.EXACT.EXPLODE("Sleep") OR TI,AB("sleep*") OR TI,AB("wake*") OR TI,AB("waking") OR TI,AB("awake"))  OR  (MAINSUBJECT.EXACT.EXPLODE("Polysomnography") OR MAINSUBJECT.EXACT.EXPLODE("Actigraphy") OR TI,AB("actigraph*") OR TI,AB("actimetr*") OR TI,AB("acceleromet*") OR TI,AB("polysomnograph*") OR TI,AB("EEG") OR TI,AB("electroencephalogram") OR TI,AB("MSLT") OR TI,AB("MWT") OR TI,AB("fitbit") OR TI,AB("dreem") OR TI,AB("Oura ring") OR TI,AB("Gen3") OR TI,AB("Fitbit") OR TI,AB("Mi band"))  OR  (MAINSUBJECT.EXACT.EXPLODE("Human Biological Rhythms") OR TI,AB("circadian") OR TI,AB("chronotype*") OR TI,AB("chronotherap*") OR TI,AB("eveningness") OR TI,AB("morningness") OR TI,AB("evening type*") OR TI,AB("morning type*") OR TI,AB("bedtime*") OR TI,AB("time to bed") OR TI,AB("time in bed") OR TI,AB("shuteye") OR TI,AB("shut-eye") OR TI,AB("lights off") OR TI,AB("lights on") OR MAINSUBJECT.EXACT("Workday Shifts") OR TI,AB("shift work*") OR TI,AB("shiftwork*") OR TI,AB("shift" NEAR/2 "schedule") OR TI,AB("shift" NEAR/2 "schedules") OR TI,AB("shift" NEAR/2 "scheduling") OR TI,AB("shifting" NEAR/2 "schedule") OR TI,AB("shifting" NEAR/2 "schedules") OR TI,AB("shifting" NEAR/2 "scheduling") OR TI,AB("working" NEAR/2 "hours") OR TI,AB("work" NEAR/2 "hours") OR TI,AB("work" NEAR/2 "schedule") OR TI,AB("work" NEAR/2 "schedules") OR TI,AB("work" NEAR/2 "scheduling") OR TI,AB("working" NEAR/2 "schedule") OR TI,AB("working" NEAR/2 "schedules") OR TI,AB("jetlag") OR TI,AB("jet-lag") OR TI,AB("light*") OR TI,AB("nois*") OR TI,AB("WASO") OR TI,AB("TIB") OR TI,AB("SE"))  OR  (MAINSUBJECT.EXACT.EXPLODE("Sleepiness") OR TI,AB("fatigue*") OR TI,AB("tired*") OR TI,AB("somnolence") OR TI,AB("nap") OR TI,AB("napping") OR TI,AB("alert*") OR TI,AB("ESS") OR TI,AB("KSS") OR TI,AB("EDS") OR TI,AB("day") OR TI,AB("daytime") OR TI,AB("night*") OR TI,AB("drows*") OR TI,AB("siesta"))  OR  (MAINSUBJECT.EXACT.EXPLODE("Sleep Wake Disorders") OR TI,AB("insomnia") OR TI,AB("restless legs syndrome") OR TI,AB("restless leg syndrome") OR TI,AB("Willis-Ekbom") OR TI,AB("Wittmaack-Ekbom") OR TI,AB("RLS") OR TI,AB("periodic leg movement*") OR TI,AB("periodic limb movement*") OR MAINSUBJECT.EXACT.EXPLODE("Snoring") OR TI,AB("snoring") OR TI,AB("snore") OR TI,AB("hypersomnia") OR TI,AB("dyssomnia") OR TI,AB("parasomnia") OR TI,AB("narcolepsy") OR TI,AB("night terror") OR TI,AB("nightmare*") OR MAINSUBJECT.EXACT.EXPLODE("Apnea") OR TI,AB("apnea") OR TI,AB("apnoea") OR TI,AB("hypopnea") OR TI,AB("hypopnea") OR TI,AB("OSA") OR TI,AB("OSAHS") OR TI,AB("AHI") OR TI,AB("CSA") OR TI,AB("UARS") OR TI,AB("upper airway resistance syndrome"))  **)** |
| **3** |  | Multidimensionality | **(**  (MAINSUBJECT.EXACT.EXPLODE("Sleep") OR TI,AB("sleep*"))  AND  (  (TI,AB("RU-SATED") OR TI,AB("RU_SATED") OR TI,AB("PSQI"))  OR  (TI,AB("score*") OR TI,AB("index") OR TI,AB("indices") OR TI,AB("multidimensional") OR TI,AB("multi-dimensional") OR TI,AB("multi*") OR TI,AB("dimension*") OR TI,AB("component*") OR TI,AB("parameter*") OR TI,AB("metric*") OR TI,AB("composite") OR TI,AB("combination*"))  )  **)** |
| **4** |  | Multidimensional sleep health | **(#1** AND **(#2** OR **#3))** |
| **5** |  | All-cause outcomes | **(**MAINSUBJECT.EXACT.EXPLODE("Death and Dying") OR TI,AB("mortality") OR TI,AB("death*") OR TI,AB("lethal")**)** |
| **6** |  | Specific outcomes of interest | **(**  (TI,AB("SCD") OR TI,AB("cardiopulmonary arrest") OR TI,AB("cardiac arrest") OR TI,AB("heart arrest") OR TI,AB("asystole") OR TI,AB("cardiac event*") OR MAINSUBJECT.EXACT.EXPLODE("Myocardial Infarctions") OR TI,AB("myocardial infarct*") OR TI,AB("myocardial ischemia") OR TI,AB("unstable angina") OR TI,AB("acute coronary syndrome") OR TI,AB("ACS") OR TI,AB("AMI") OR TI,AB("MI") OR TI,AB("heart failure") OR TI,AB("cardiac failure") OR TI,AB("myocardial failure") OR TI,AB("heart decompensation") OR TI,AB("ventricular dysfunction") OR TI,AB("CHF"))  OR  (MAINSUBJECT.EXACT.EXPLODE("Cerebrovascular Accidents") OR TI,AB("stroke") OR TI,AB("cerebral infarct*") OR TI,AB("cerebrovascular accident*") OR TI,AB("CVA") OR TI,AB("brain vascular accident*") OR TI,AB("cerebrovascular apoplexy") OR TI,AB("brain ischemia") OR TI,AB("intracranial hemorrhage") OR TI,AB("intracranial haemorrhage") OR TI,AB("cerebral hemorrhage") OR TI,AB("cerebral haemorrhage"))  OR  (TI,AB("MACE") OR TI,AB("major adverse cardiovascular event*") OR TI,AB("infarct*"))  **)** |
| **7** |  | Outcomes | **(#5** OR **#6)** |
| **8** |  | Full query | **#4** AND **#7**  (( (MAINSUBJECT.EXACT.EXPLODE("Sleep Deprivation") OR TI,AB("sleep deprivation") OR TI,AB("insufficient sleep")) OR (TI,AB("quantity" NEAR/2 "sleep") OR TI,AB("quantities" NEAR/2 "sleep") OR TI,AB("amount" NEAR/2 "sleep") OR TI,AB("duration" NEAR/2 "sleep") OR TI,AB("length" NEAR/2 "sleep") OR TI,AB("time" NEAR/2 "sleep") OR TI,AB("period" NEAR/2 "sleep") OR TI,AB("hours" NEAR/2 "sleep") OR TI,AB("minutes" NEAR/2 "sleep") OR TI,AB("span" NEAR/2 "sleep")) OR (TI,AB("duration" NEAR/2 "asleep") OR TI,AB("length" NEAR/2 "asleep") OR TI,AB("time" NEAR/2 "asleep") OR TI,AB("period" NEAR/2 "asleep") OR TI,AB("hours" NEAR/2 "asleep") OR TI,AB("minutes" NEAR/2 "asleep")) OR (TI,AB("quantity" NEAR/2 "sleeping") OR TI,AB("quantities" NEAR/2 "sleeping") OR TI,AB("amount" NEAR/2 "sleeping") OR TI,AB("duration" NEAR/2 "sleeping") OR TI,AB("length" NEAR/2 "sleeping") OR TI,AB("time" NEAR/2 "sleeping") OR TI,AB("period" NEAR/2 "sleeping") OR TI,AB("hours" NEAR/2 "sleeping") OR TI,AB("minutes" NEAR/2 "sleeping") OR TI,AB("span" NEAR/2 "sleeping")) OR (TI,AB("short" NEAR/2 "sleep") OR TI,AB("long" NEAR/2 "sleep") OR TI,AB("extended" NEAR/2 "sleep")) OR (TI,AB("short" NEAR/2 "sleeper") OR TI,AB("long" NEAR/2 "sleeper") OR TI,AB("extended" NEAR/2 "sleeper")) OR (TI,AB("short" NEAR/2 "sleepers") OR TI,AB("long" NEAR/2 "sleepers") OR TI,AB("extended" NEAR/2 "sleepers")) ) AND (( (MAINSUBJECT.EXACT.EXPLODE("Sleep") OR TI,AB("sleep*") OR TI,AB("wake*") OR TI,AB("waking") OR TI,AB("awake")) OR (MAINSUBJECT.EXACT.EXPLODE("Polysomnography") OR MAINSUBJECT.EXACT.EXPLODE("Actigraphy") OR TI,AB("actigraph*") OR TI,AB("actimetr*") OR TI,AB("acceleromet*") OR TI,AB("polysomnograph*") OR TI,AB("EEG") OR TI,AB("electroencephalogram") OR TI,AB("MSLT") OR TI,AB("MWT") OR TI,AB("fitbit") OR TI,AB("dreem") OR TI,AB("Oura ring") OR TI,AB("Gen3") OR TI,AB("Fitbit") OR TI,AB("Mi band")) OR (MAINSUBJECT.EXACT.EXPLODE("Human Biological Rhythms") OR TI,AB("circadian") OR TI,AB("chronotype*") OR TI,AB("chronotherap*") OR TI,AB("eveningness") OR TI,AB("morningness") OR TI,AB("evening type*") OR TI,AB("morning type*") OR TI,AB("bedtime*") OR TI,AB("time to bed") OR TI,AB("time in bed") OR TI,AB("shuteye") OR TI,AB("shut-eye") OR TI,AB("lights off") OR TI,AB("lights on") OR MAINSUBJECT.EXACT("Workday Shifts") OR TI,AB("shift work*") OR TI,AB("shiftwork*") OR TI,AB("shift" NEAR/2 "schedule") OR TI,AB("shift" NEAR/2 "schedules") OR TI,AB("shift" NEAR/2 "scheduling") OR TI,AB("shifting" NEAR/2 "schedule") OR TI,AB("shifting" NEAR/2 "schedules") OR TI,AB("shifting" NEAR/2 "scheduling") OR TI,AB("working" NEAR/2 "hours") OR TI,AB("work" NEAR/2 "hours") OR TI,AB("work" NEAR/2 "schedule") OR TI,AB("work" NEAR/2 "schedules") OR TI,AB("work" NEAR/2 "scheduling") OR TI,AB("working" NEAR/2 "schedule") OR TI,AB("working" NEAR/2 "schedules") OR TI,AB("jetlag") OR TI,AB("jet-lag") OR TI,AB("light*") OR TI,AB("nois*") OR TI,AB("WASO") OR TI,AB("TIB") OR TI,AB("SE")) OR (MAINSUBJECT.EXACT.EXPLODE("Sleepiness") OR TI,AB("fatigue*") OR TI,AB("tired*") OR TI,AB("somnolence") OR TI,AB("nap") OR TI,AB("napping") OR TI,AB("alert*") OR TI,AB("ESS") OR TI,AB("KSS") OR TI,AB("EDS") OR TI,AB("day") OR TI,AB("daytime") OR TI,AB("night*") OR TI,AB("drows*") OR TI,AB("siesta")) OR (MAINSUBJECT.EXACT.EXPLODE("Sleep Wake Disorders") OR TI,AB("insomnia") OR TI,AB("restless legs syndrome") OR TI,AB("restless leg syndrome") OR TI,AB("Willis-Ekbom") OR TI,AB("Wittmaack-Ekbom") OR TI,AB("RLS") OR TI,AB("periodic leg movement*") OR TI,AB("periodic limb movement*") OR MAINSUBJECT.EXACT.EXPLODE("Snoring") OR TI,AB("snoring") OR TI,AB("snore") OR TI,AB("hypersomnia") OR TI,AB("dyssomnia") OR TI,AB("parasomnia") OR TI,AB("narcolepsy") OR TI,AB("night terror") OR TI,AB("nightmare*") OR MAINSUBJECT.EXACT.EXPLODE("Apnea") OR TI,AB("apnea") OR TI,AB("apnoea") OR TI,AB("hypopnea") OR TI,AB("hypopnea") OR TI,AB("OSA") OR TI,AB("OSAHS") OR TI,AB("AHI") OR TI,AB("CSA") OR TI,AB("UARS") OR TI,AB("upper airway resistance syndrome")) ) OR ( (MAINSUBJECT.EXACT.EXPLODE("Sleep") OR TI,AB("sleep*")) AND ( (TI,AB("RU-SATED") OR TI,AB("RU_SATED") OR TI,AB("PSQI")) OR (TI,AB("score*") OR TI,AB("index") OR TI,AB("indices") OR TI,AB("multidimensional") OR TI,AB("multi-dimensional") OR TI,AB("multi*") OR TI,AB("dimension*") OR TI,AB("component*") OR TI,AB("parameter*") OR TI,AB("metric*") OR TI,AB("composite") OR TI,AB("combination*")) ) ) )) AND ((MAINSUBJECT.EXACT.EXPLODE("Death and Dying") OR TI,AB("mortality") OR TI,AB("death*") OR TI,AB("lethal")) OR ( (TI,AB("SCD") OR TI,AB("cardiopulmonary arrest") OR TI,AB("cardiac arrest") OR TI,AB("heart arrest") OR TI,AB("asystole") OR TI,AB("cardiac event*") OR MAINSUBJECT.EXACT.EXPLODE("Myocardial Infarctions") OR TI,AB("myocardial infarct*") OR TI,AB("myocardial ischemia") OR TI,AB("unstable angina") OR TI,AB("acute coronary syndrome") OR TI,AB("ACS") OR TI,AB("AMI") OR TI,AB("MI") OR TI,AB("heart failure") OR TI,AB("cardiac failure") OR TI,AB("myocardial failure") OR TI,AB("heart decompensation") OR TI,AB("ventricular dysfunction") OR TI,AB("CHF")) OR (MAINSUBJECT.EXACT.EXPLODE("Cerebrovascular Accidents") OR TI,AB("stroke") OR TI,AB("cerebral infarct*") OR TI,AB("cerebrovascular accident*") OR TI,AB("CVA") OR TI,AB("brain vascular accident*") OR TI,AB("cerebrovascular apoplexy") OR TI,AB("brain ischemia") OR TI,AB("intracranial hemorrhage") OR TI,AB("intracranial haemorrhage") OR TI,AB("cerebral hemorrhage") OR TI,AB("cerebral haemorrhage")) OR (TI,AB("MACE") OR TI,AB("major adverse cardiovascular event*") OR TI,AB("infarct*")) ) ) |

**Abbreviations.** MAINSUBJECT.EXACT.EXPLODE: controlled vocabulary search including underlying subject headings. NEAR/2: search for word before and after in any order with up to two words in-between (proximity parameter). TI,AB: search to be made in the title and abstract fields. **Clarifications.** 1) **bold font-weight** visually indicates blocks of search terms or parentheses encapsulating these; 2) **green color** denotes blocks of search terms; 3) dark blue color denotes search terms with the TI,AB field setting without the proximity parameter; 4) light blue color denotes search terms with the TI,AB field setting with the proximity parameter; 5) purple color denotes controlled vocabulary search terms.

## 6. PubMed

| **#** | **Block name** | **Search terms** |
| --- | --- | --- |
| **1** | Sleep duration | **(**  ("Sleep Duration"[mh] OR " Sleep Deprivation"[mh] OR "sleep deprivation"[tiab] OR "insufficient sleep"[tiab])  OR  ("quantity sleep"[tiab:~2] OR "quantities sleep"[tiab:~2] OR "amount sleep"[tiab:~2] OR "duration sleep"[tiab:~2] OR "length sleep"[tiab:~2] OR "time sleep"[tiab:~2] OR "period sleep"[tiab:~2] OR "hours sleep"[tiab:~2] OR "minutes sleep"[tiab:~2] OR "span sleep"[tiab:~2])  OR  ("duration asleep"[tiab:~2] OR "length asleep"[tiab:~2] OR "time asleep"[tiab:~2] OR "period asleep"[tiab:~2] OR "hours asleep"[tiab:~2] OR "minutes asleep"[tiab:~2])  OR  ("quantity sleeping"[tiab:~2] OR "quantities sleeping"[tiab:~2] OR "amount sleeping"[tiab:~2] OR "duration sleeping"[tiab:~2] OR "length sleeping"[tiab:~2] OR "time sleeping"[tiab:~2] OR "period sleeping"[tiab:~2] OR "hours sleeping"[tiab:~2] OR "minutes sleeping"[tiab:~2] OR "span sleeping"[tiab:~2])  OR  ("short sleep"[tiab:~2] OR "long sleep"[tiab:~2] OR "extended sleep"[tiab:~2])  OR  ("short sleeper"[tiab:~2] OR "long sleeper"[tiab:~2] OR "extended sleeper"[tiab:~2])  OR  ("short sleepers"[tiab:~2] OR "long sleepers"[tiab:~2] OR "extended sleepers"[tiab:~2])  **)** |
| **2** | Sleep components | **(**  ("Sleep"[mh] OR "sleep*"[tiab] OR "wake*"[tiab] OR "waking"[tiab] OR "awake"[tiab])  OR  ("Polysomnography"[mh] OR "Actigraphy"[mh] OR "actigraph*"[tiab] OR "actimetr*"[tiab] OR "acceleromet*"[tiab] OR "polysomnograph*"[tiab] OR "EEG"[tiab] OR "electroencephalogram"[tiab] OR "MSLT"[tiab] OR "MWT"[tiab] OR "fitbit"[tiab] OR "dreem"[tiab] OR "Oura ring"[tiab] OR "Gen3"[tiab] OR "Fitbit"[tiab] OR "Mi band"[tiab])  OR  ("Circadian Clocks"[mh] OR "Circadian Rhythm"[mh] OR "circadian"[tiab] OR "chronotype*"[tiab] OR "chronotherap*"[tiab] OR "eveningness"[tiab] OR "morningness"[tiab] OR "evening type*"[tiab] OR "morning type*"[tiab] OR "bedtime*"[tiab] OR "time to bed"[tiab] OR "time in bed"[tiab] OR "shuteye"[tiab] OR "shut-eye"[tiab] OR "lights off"[tiab] OR "lights on"[tiab] OR "Shift Work Schedule"[mh] OR "shift work*"[tiab] OR "shiftwork*"[tiab] OR "shift schedule"[tiab:~2] OR "shift schedules"[tiab:~2] OR "shift scheduling"[tiab:~2] OR "shifting schedule"[tiab:~2] OR "shifting schedules"[tiab:~2] OR "shifting scheduling"[tiab:~2] OR "working hours"[tiab:~2] OR "work hours"[tiab:~2] OR "work schedule"[tiab:~2] OR "work schedules"[tiab:~2] OR "work scheduling"[tiab:~2] OR "working schedule"[tiab:~2] OR "working schedules"[tiab:~2] OR "jetlag"[tiab] OR "jet-lag"[tiab] OR "light*"[tiab] OR "nois*"[tiab] OR "WASO"[tiab] OR "TIB"[tiab] OR "SE"[tiab])  OR  ("Sleepiness"[mh] OR "fatigue*"[tiab] OR "tired*"[tiab] OR "somnolence"[tiab] OR "nap"[tiab] OR "napping"[tiab] OR "alert*"[tiab] OR "ESS"[tiab] OR "KSS"[tiab] OR "EDS"[tiab] OR "day"[tiab] OR "daytime"[tiab] OR "night*"[tiab] OR "drows*"[tiab] OR "siesta"[tiab])  OR  ("Sleep Wake Disorders"[mh] OR "insomnia"[tiab] OR "restless legs syndrome"[tiab] OR "restless leg syndrome"[tiab] OR "Willis-Ekbom"[tiab] OR "Wittmaack-Ekbom"[tiab] OR "RLS"[tiab] OR "periodic leg movement*"[tiab] OR "periodic limb movement*"[tiab] OR "Snoring"[mh] OR "snoring"[tiab] OR "snore"[tiab] OR "hypersomnia"[tiab] OR "dyssomnia"[tiab] OR "parasomnia"[tiab] OR "narcolepsy"[tiab] OR "night terror"[tiab] OR "nightmare*"[tiab] OR "Apnea"[mh] OR "apnea"[tiab] OR "apnoea"[tiab] OR "hypopnea"[tiab] OR "hypopnea"[tiab] OR "OSA"[tiab] OR "OSAHS"[tiab] OR "AHI"[tiab] OR "CSA"[tiab] OR "UARS"[tiab] OR "upper airway resistance syndrome"[tiab])  **)** |
| **3** | Multidimensionality | **(**  ("Sleep"[mh] OR "sleep*"[tiab])  AND  (  ("RU-SATED"[tiab] OR "RU_SATED"[tiab] OR "PSQI"[tiab])  OR  ("score*"[tiab] OR "index"[tiab] OR "indices"[tiab] OR "multidimensional"[tiab] OR "multi-dimensional"[tiab] OR "multi*"[tiab] OR "dimension*"[tiab] OR "component*"[tiab] OR "parameter*"[tiab] OR "metric*"[tiab] OR "composite"[tiab] OR "combination*"[tiab])  )  **)** |
| **4** | Multidimensional sleep health | **(#1** AND **(#2** OR **#3))** |
| **5** | All-cause outcomes | **(**"Mortality"[mh] OR "mortality"[tiab] OR "death*"[tiab] OR "lethal"[tiab]**)** |
| **6** | Specific outcomes of interest | **(**  ("Heart Arrest"[mh] OR "SCD"[tiab] OR "cardiopulmonary arrest"[tiab] OR "cardiac arrest"[tiab] OR "heart arrest"[tiab] OR "asystole"[tiab] OR "cardiac event*"[tiab] OR "Myocardial Infarction"[mh] OR "myocardial infarct*"[tiab] OR "Myocardial Ischemia"[mh] OR "myocardial ischemia"[tiab] OR "unstable angina"[tiab] OR "acute coronary syndrome"[tiab] OR "ACS"[tiab] OR "AMI"[tiab] OR "MI"[tiab] OR "Heart Failure"[mh] OR "heart failure"[tiab] OR "cardiac failure"[tiab] OR "myocardial failure"[tiab] OR "heart decompensation"[tiab] OR "ventricular dysfunction"[tiab] OR "CHF"[tiab])  OR  ("Stroke"[mh] OR "stroke"[tiab] OR "cerebral infarct*"[tiab] OR "cerebrovascular accident*"[tiab] OR "CVA"[tiab] OR "brain vascular accident*"[tiab] OR "cerebrovascular apoplexy"[tiab] OR "brain ischemia"[tiab] OR "intracranial hemorrhage"[tiab] OR "intracranial haemorrhage"[tiab] OR "cerebral hemorrhage"[tiab] OR "cerebral haemorrhage"[tiab])  OR  ("MACE"[tiab] OR "major adverse cardiovascular event*"[tiab] OR "infarct*"[tiab])  **)** |
| **7** | Outcomes | **(#5** OR **#6)** |
| **8** | Full query | **#4** AND **#7**  (( ("Sleep Duration"[mh] OR " Sleep Deprivation"[mh] OR "sleep deprivation"[tiab] OR "insufficient sleep"[tiab]) OR ("quantity sleep"[tiab:~2] OR "quantities sleep"[tiab:~2] OR "amount sleep"[tiab:~2] OR "duration sleep"[tiab:~2] OR "length sleep"[tiab:~2] OR "time sleep"[tiab:~2] OR "period sleep"[tiab:~2] OR "hours sleep"[tiab:~2] OR "minutes sleep"[tiab:~2] OR "span sleep"[tiab:~2]) OR ("duration asleep"[tiab:~2] OR "length asleep"[tiab:~2] OR "time asleep"[tiab:~2] OR "period asleep"[tiab:~2] OR "hours asleep"[tiab:~2] OR "minutes asleep"[tiab:~2]) OR ("quantity sleeping"[tiab:~2] OR "quantities sleeping"[tiab:~2] OR "amount sleeping"[tiab:~2] OR "duration sleeping"[tiab:~2] OR "length sleeping"[tiab:~2] OR "time sleeping"[tiab:~2] OR "period sleeping"[tiab:~2] OR "hours sleeping"[tiab:~2] OR "minutes sleeping"[tiab:~2] OR "span sleeping"[tiab:~2]) OR ("short sleep"[tiab:~2] OR "long sleep"[tiab:~2] OR "extended sleep"[tiab:~2]) OR ("short sleeper"[tiab:~2] OR "long sleeper"[tiab:~2] OR "extended sleeper"[tiab:~2]) OR ("short sleepers"[tiab:~2] OR "long sleepers"[tiab:~2] OR "extended sleepers"[tiab:~2]) ) AND (( ("Sleep"[mh] OR "sleep*"[tiab] OR "wake*"[tiab] OR "waking"[tiab] OR "awake"[tiab]) OR ("Polysomnography"[mh] OR "Actigraphy"[mh] OR "actigraph*"[tiab] OR "actimetr*"[tiab] OR "acceleromet*"[tiab] OR "polysomnograph*"[tiab] OR "EEG"[tiab] OR "electroencephalogram"[tiab] OR "MSLT"[tiab] OR "MWT"[tiab] OR "fitbit"[tiab] OR "dreem"[tiab] OR "Oura ring"[tiab] OR "Gen3"[tiab] OR "Fitbit"[tiab] OR "Mi band"[tiab]) OR ("Circadian Clocks"[mh] OR "Circadian Rhythm"[mh] OR "circadian"[tiab] OR "chronotype*"[tiab] OR "chronotherap*"[tiab] OR "eveningness"[tiab] OR "morningness"[tiab] OR "evening type*"[tiab] OR "morning type*"[tiab] OR "bedtime*"[tiab] OR "time to bed"[tiab] OR "time in bed"[tiab] OR "shuteye"[tiab] OR "shut-eye"[tiab] OR "lights off"[tiab] OR "lights on"[tiab] OR "Shift Work Schedule"[mh] OR "shift work*"[tiab] OR "shiftwork*"[tiab] OR "shift schedule"[tiab:~2] OR "shift schedules"[tiab:~2] OR "shift scheduling"[tiab:~2] OR "shifting schedule"[tiab:~2] OR "shifting schedules"[tiab:~2] OR "shifting scheduling"[tiab:~2] OR "working hours"[tiab:~2] OR "work hours"[tiab:~2] OR "work schedule"[tiab:~2] OR "work schedules"[tiab:~2] OR "work scheduling"[tiab:~2] OR "working schedule"[tiab:~2] OR "working schedules"[tiab:~2] OR "jetlag"[tiab] OR "jet-lag"[tiab] OR "light*"[tiab] OR "nois*"[tiab] OR "WASO"[tiab] OR "TIB"[tiab] OR "SE"[tiab]) OR ("Sleepiness"[mh] OR "fatigue*"[tiab] OR "tired*"[tiab] OR "somnolence"[tiab] OR "nap"[tiab] OR "napping"[tiab] OR "alert*"[tiab] OR "ESS"[tiab] OR "KSS"[tiab] OR "EDS"[tiab] OR "day"[tiab] OR "daytime"[tiab] OR "night*"[tiab] OR "drows*"[tiab] OR "siesta"[tiab]) OR ("Sleep Wake Disorders"[mh] OR "insomnia"[tiab] OR "restless legs syndrome"[tiab] OR "restless leg syndrome"[tiab] OR "Willis-Ekbom"[tiab] OR "Wittmaack-Ekbom"[tiab] OR "RLS"[tiab] OR "periodic leg movement*"[tiab] OR "periodic limb movement*"[tiab] OR "Snoring"[mh] OR "snoring"[tiab] OR "snore"[tiab] OR "hypersomnia"[tiab] OR "dyssomnia"[tiab] OR "parasomnia"[tiab] OR "narcolepsy"[tiab] OR "night terror"[tiab] OR "nightmare*"[tiab] OR "Apnea"[mh] OR "apnea"[tiab] OR "apnoea"[tiab] OR "hypopnea"[tiab] OR "hypopnea"[tiab] OR "OSA"[tiab] OR "OSAHS"[tiab] OR "AHI"[tiab] OR "CSA"[tiab] OR "UARS"[tiab] OR "upper airway resistance syndrome"[tiab]) ) OR ( ("Sleep"[mh] OR "sleep*"[tiab]) AND ( ("RU-SATED"[tiab] OR "RU_SATED"[tiab] OR "PSQI"[tiab]) OR ("score*"[tiab] OR "index"[tiab] OR "indices"[tiab] OR "multidimensional"[tiab] OR "multi-dimensional"[tiab] OR "multi*"[tiab] OR "dimension*"[tiab] OR "component*"[tiab] OR "parameter*"[tiab] OR "metric*"[tiab] OR "composite"[tiab] OR "combination*"[tiab]) ) ) )) AND (("Mortality"[mh] OR "mortality"[tiab] OR "death*"[tiab] OR "lethal"[tiab]) OR ( ("Heart Arrest"[mh] OR "SCD"[tiab] OR "cardiopulmonary arrest"[tiab] OR "cardiac arrest"[tiab] OR "heart arrest"[tiab] OR "asystole"[tiab] OR "cardiac event*"[tiab] OR "Myocardial Infarction"[mh] OR "myocardial infarct*"[tiab] OR "Myocardial Ischemia"[mh] OR "myocardial ischemia"[tiab] OR "unstable angina"[tiab] OR "acute coronary syndrome"[tiab] OR "ACS"[tiab] OR "AMI"[tiab] OR "MI"[tiab] OR "Heart Failure"[mh] OR "heart failure"[tiab] OR "cardiac failure"[tiab] OR "myocardial failure"[tiab] OR "heart decompensation"[tiab] OR "ventricular dysfunction"[tiab] OR "CHF"[tiab]) OR ("Stroke"[mh] OR "stroke"[tiab] OR "cerebral infarct*"[tiab] OR "cerebrovascular accident*"[tiab] OR "CVA"[tiab] OR "brain vascular accident*"[tiab] OR "cerebrovascular apoplexy"[tiab] OR "brain ischemia"[tiab] OR "intracranial hemorrhage"[tiab] OR "intracranial haemorrhage"[tiab] OR "cerebral hemorrhage"[tiab] OR "cerebral haemorrhage"[tiab]) OR ("MACE"[tiab] OR "major adverse cardiovascular event*"[tiab] OR "infarct*"[tiab]) ) ) |

**Abbreviations.** [mh]: search to be made by Medical Subject Headings (MeSH), a controlled vocabulary. [tiab]: search to be made in title and abstract fields (search terms followed by ":~2" in the field setting denote search terms with a proximity parameter [in which the two words are allowed to occur in any order with up to two words in-between]). **Clarifications.** 1) **bold font-weight** visually indicates blocks of search terms or parentheses encapsulating these; 2) **green color** denotes blocks of search terms; 3) dark blue color denotes search terms with the [tiab] field setting without the proximity parameter; 4) light blue color denotes search terms with the [tiab] field setting with the proximity parameter; 5) purple color denotes controlled vocabulary search terms.

## 7. Scopus

| **#** | **Block name** | **Search terms** |
| --- | --- | --- |
| **1** | Sleep duration | **(**  (TITLE-ABS-KEY("sleep deprivation") OR TITLE-ABS-KEY("insufficient sleep"))  OR  (TITLE-ABS-KEY("quantity" w/2 "sleep") OR TITLE-ABS-KEY("quantities" w/2 "sleep") OR TITLE-ABS-KEY("amount" w/2 "sleep") OR TITLE-ABS-KEY("duration" w/2 "sleep") OR TITLE-ABS-KEY("length" w/2 "sleep") OR TITLE-ABS-KEY("time" w/2 "sleep") OR TITLE-ABS-KEY("period" w/2 "sleep") OR TITLE-ABS-KEY("hours" w/2 "sleep") OR TITLE-ABS-KEY("minutes" w/2 "sleep") OR TITLE-ABS-KEY("span" w/2 "sleep"))  OR  (TITLE-ABS-KEY("duration" w/2 "asleep") OR TITLE-ABS-KEY("length" w/2 "asleep") OR TITLE-ABS-KEY("time" w/2 "asleep") OR TITLE-ABS-KEY("period" w/2 "asleep") OR TITLE-ABS-KEY("hours" w/2 "asleep") OR TITLE-ABS-KEY("minutes" w/2 "asleep"))  OR  (TITLE-ABS-KEY("quantity" w/2 "sleeping") OR TITLE-ABS-KEY("quantities" w/2 "sleeping") OR TITLE-ABS-KEY("amount" w/2 "sleeping") OR TITLE-ABS-KEY("duration" w/2 "sleeping") OR TITLE-ABS-KEY("length" w/2 "sleeping") OR TITLE-ABS-KEY("time" w/2 "sleeping") OR TITLE-ABS-KEY("period" w/2 "sleeping") OR TITLE-ABS-KEY("hours" w/2 "sleeping") OR TITLE-ABS-KEY("minutes" w/2 "sleeping") OR TITLE-ABS-KEY("span" w/2 "sleeping"))  OR  (TITLE-ABS-KEY("short" w/2 "sleep") OR TITLE-ABS-KEY("long" w/2 "sleep") OR TITLE-ABS-KEY("extended" w/2 "sleep"))  OR  (TITLE-ABS-KEY("short" w/2 "sleeper") OR TITLE-ABS-KEY("long" w/2 "sleeper") OR TITLE-ABS-KEY("extended" w/2 "sleeper"))  OR  (TITLE-ABS-KEY("short" w/2 "sleepers") OR TITLE-ABS-KEY("long" w/2 "sleepers") OR TITLE-ABS-KEY("extended" w/2 "sleepers"))  **)** |
| **2** | Sleep components | **(**  (TITLE-ABS-KEY("sleep*") OR TITLE-ABS-KEY("wake*") OR TITLE-ABS-KEY("waking") OR TITLE-ABS-KEY("awake"))  OR  (TITLE-ABS-KEY("actigraph*") OR TITLE-ABS-KEY("actimetr*") OR TITLE-ABS-KEY("acceleromet*") OR TITLE-ABS-KEY("polysomnograph*") OR TITLE-ABS-KEY("EEG") OR TITLE-ABS-KEY("electroencephalogram") OR TITLE-ABS-KEY("MSLT") OR TITLE-ABS-KEY("MWT") OR TITLE-ABS-KEY("fitbit") OR TITLE-ABS-KEY("dreem") OR TITLE-ABS-KEY("Oura ring") OR TITLE-ABS-KEY("Gen3") OR TITLE-ABS-KEY("Fitbit") OR TITLE-ABS-KEY("Mi band"))  OR  (TITLE-ABS-KEY("circadian") OR TITLE-ABS-KEY("chronotype*") OR TITLE-ABS-KEY("chronotherap*") OR TITLE-ABS-KEY("eveningness") OR TITLE-ABS-KEY("morningness") OR TITLE-ABS-KEY("evening type*") OR TITLE-ABS-KEY("morning type*") OR TITLE-ABS-KEY("bedtime*") OR TITLE-ABS-KEY("time to bed") OR TITLE-ABS-KEY("time in bed") OR TITLE-ABS-KEY("shuteye") OR TITLE-ABS-KEY("shut-eye") OR TITLE-ABS-KEY("lights off") OR TITLE-ABS-KEY("lights on") OR TITLE-ABS-KEY("shift work*") OR TITLE-ABS-KEY("shiftwork*") OR TITLE-ABS-KEY("shift" w/2 "schedule") OR TITLE-ABS-KEY("shift" w/2 "schedules") OR TITLE-ABS-KEY("shift" w/2 "scheduling") OR TITLE-ABS-KEY("shifting" w/2 "schedule") OR TITLE-ABS-KEY("shifting" w/2 "schedules") OR TITLE-ABS-KEY("shifting" w/2 "scheduling") OR TITLE-ABS-KEY("working" w/2 "hours") OR TITLE-ABS-KEY("work" w/2 "hours") OR TITLE-ABS-KEY("work" w/2 "schedule") OR TITLE-ABS-KEY("work" w/2 "schedules") OR TITLE-ABS-KEY("work" w/2 "scheduling") OR TITLE-ABS-KEY("working" w/2 "schedule") OR TITLE-ABS-KEY("working" w/2 "schedules") OR TITLE-ABS-KEY("jetlag") OR TITLE-ABS-KEY("jet-lag") OR TITLE-ABS-KEY("light*") OR TITLE-ABS-KEY("nois*") OR TITLE-ABS-KEY("WASO") OR TITLE-ABS-KEY("TIB") OR TITLE-ABS-KEY("SE"))  OR  (TITLE-ABS-KEY("fatigue*") OR TITLE-ABS-KEY("tired*") OR TITLE-ABS-KEY("somnolence") OR TITLE-ABS-KEY("nap") OR TITLE-ABS-KEY("napping") OR TITLE-ABS-KEY("alert*") OR TITLE-ABS-KEY("ESS") OR TITLE-ABS-KEY("KSS") OR TITLE-ABS-KEY("EDS") OR TITLE-ABS-KEY("day") OR TITLE-ABS-KEY("daytime") OR TITLE-ABS-KEY("night*") OR TITLE-ABS-KEY("drows*") OR TITLE-ABS-KEY("siesta"))  OR  (TITLE-ABS-KEY("insomnia") OR TITLE-ABS-KEY("restless legs syndrome") OR TITLE-ABS-KEY("restless leg syndrome") OR TITLE-ABS-KEY("Willis-Ekbom") OR TITLE-ABS-KEY("Wittmaack-Ekbom") OR TITLE-ABS-KEY("RLS") OR TITLE-ABS-KEY("periodic leg movement*") OR TITLE-ABS-KEY("periodic limb movement*") OR TITLE-ABS-KEY("snoring") OR TITLE-ABS-KEY("snore") OR TITLE-ABS-KEY("hypersomnia") OR TITLE-ABS-KEY("dyssomnia") OR TITLE-ABS-KEY("parasomnia") OR TITLE-ABS-KEY("narcolepsy") OR TITLE-ABS-KEY("night terror") OR TITLE-ABS-KEY("nightmare*") OR TITLE-ABS-KEY("apnea") OR TITLE-ABS-KEY("apnoea") OR TITLE-ABS-KEY("hypopnea") OR TITLE-ABS-KEY("hypopnea") OR TITLE-ABS-KEY("OSA") OR TITLE-ABS-KEY("OSAHS") OR TITLE-ABS-KEY("AHI") OR TITLE-ABS-KEY("CSA") OR TITLE-ABS-KEY("UARS") OR TITLE-ABS-KEY("upper airway resistance syndrome"))  **)** |
| **3** | Multidimensionality | **(**  (TITLE-ABS-KEY("sleep*"))  AND  (  (TITLE-ABS-KEY("RU-SATED") OR TITLE-ABS-KEY("RU_SATED") OR TITLE-ABS-KEY("PSQI"))  OR  (TITLE-ABS-KEY("score*") OR TITLE-ABS-KEY("index") OR TITLE-ABS-KEY("indices") OR TITLE-ABS-KEY("multidimensional") OR TITLE-ABS-KEY("multi-dimensional") OR TITLE-ABS-KEY("multi*") OR TITLE-ABS-KEY("dimension*") OR TITLE-ABS-KEY("component*") OR TITLE-ABS-KEY("parameter*") OR TITLE-ABS-KEY("metric*") OR TITLE-ABS-KEY("composite") OR TITLE-ABS-KEY("combination*"))  )  **)** |
| **4** | Multidimensional sleep health | **(#1** AND **(#2** OR **#3))** |
| **5** | All-cause outcomes | **(**TITLE-ABS-KEY("mortality") OR TITLE-ABS-KEY("death*") OR TITLE-ABS-KEY("lethal")**)** |
| **6** | Specific outcomes of interest | **(**  (TITLE-ABS-KEY("SCD") OR TITLE-ABS-KEY("cardiopulmonary arrest") OR TITLE-ABS-KEY("cardiac arrest") OR TITLE-ABS-KEY("heart arrest") OR TITLE-ABS-KEY("asystole") OR TITLE-ABS-KEY("cardiac event*") OR TITLE-ABS-KEY("myocardial infarct*") OR TITLE-ABS-KEY("myocardial ischemia") OR TITLE-ABS-KEY("unstable angina") OR TITLE-ABS-KEY("acute coronary syndrome") OR TITLE-ABS-KEY("ACS") OR TITLE-ABS-KEY("AMI") OR TITLE-ABS-KEY("MI") OR TITLE-ABS-KEY("heart failure") OR TITLE-ABS-KEY("cardiac failure") OR TITLE-ABS-KEY("myocardial failure") OR TITLE-ABS-KEY("heart decompensation") OR TITLE-ABS-KEY("ventricular dysfunction") OR TITLE-ABS-KEY("CHF"))  OR  (TITLE-ABS-KEY("stroke") OR TITLE-ABS-KEY("cerebral infarct*") OR TITLE-ABS-KEY("cerebrovascular accident*") OR TITLE-ABS-KEY("CVA") OR TITLE-ABS-KEY("brain vascular accident*") OR TITLE-ABS-KEY("cerebrovascular apoplexy") OR TITLE-ABS-KEY("brain ischemia") OR TITLE-ABS-KEY("intracranial hemorrhage") OR TITLE-ABS-KEY("intracranial haemorrhage") OR TITLE-ABS-KEY("cerebral hemorrhage") OR TITLE-ABS-KEY("cerebral haemorrhage"))  OR  (TITLE-ABS-KEY("MACE") OR TITLE-ABS-KEY("major adverse cardiovascular event*") OR TITLE-ABS-KEY("infarct*"))  **)** |
| **7** | Outcomes | **(#5** OR **#6)** |
| **8** | Full query | **#4** AND **#7**  (( (TITLE-ABS-KEY("sleep deprivation") OR TITLE-ABS-KEY("insufficient sleep"))  OR  (TITLE-ABS-KEY("quantity" w/2 "sleep") OR TITLE-ABS-KEY("quantities" w/2 "sleep") OR TITLE-ABS-KEY("amount" w/2 "sleep") OR TITLE-ABS-KEY("duration" w/2 "sleep") OR TITLE-ABS-KEY("length" w/2 "sleep") OR TITLE-ABS-KEY("time" w/2 "sleep") OR TITLE-ABS-KEY("period" w/2 "sleep") OR TITLE-ABS-KEY("hours" w/2 "sleep") OR TITLE-ABS-KEY("minutes" w/2 "sleep") OR TITLE-ABS-KEY("span" w/2 "sleep"))  OR  (TITLE-ABS-KEY("duration" w/2 "asleep") OR TITLE-ABS-KEY("length" w/2 "asleep") OR TITLE-ABS-KEY("time" w/2 "asleep") OR TITLE-ABS-KEY("period" w/2 "asleep") OR TITLE-ABS-KEY("hours" w/2 "asleep") OR TITLE-ABS-KEY("minutes" w/2 "asleep"))  OR  (TITLE-ABS-KEY("quantity" w/2 "sleeping") OR TITLE-ABS-KEY("quantities" w/2 "sleeping") OR TITLE-ABS-KEY("amount" w/2 "sleeping") OR TITLE-ABS-KEY("duration" w/2 "sleeping") OR TITLE-ABS-KEY("length" w/2 "sleeping") OR TITLE-ABS-KEY("time" w/2 "sleeping") OR TITLE-ABS-KEY("period" w/2 "sleeping") OR TITLE-ABS-KEY("hours" w/2 "sleeping") OR TITLE-ABS-KEY("minutes" w/2 "sleeping") OR TITLE-ABS-KEY("span" w/2 "sleeping"))  OR  (TITLE-ABS-KEY("short" w/2 "sleep") OR TITLE-ABS-KEY("long" w/2 "sleep") OR TITLE-ABS-KEY("extended" w/2 "sleep"))  OR  (TITLE-ABS-KEY("short" w/2 "sleeper") OR TITLE-ABS-KEY("long" w/2 "sleeper") OR TITLE-ABS-KEY("extended" w/2 "sleeper"))  OR  (TITLE-ABS-KEY("short" w/2 "sleepers") OR TITLE-ABS-KEY("long" w/2 "sleepers") OR TITLE-ABS-KEY("extended" w/2 "sleepers")) )  AND (( (TITLE-ABS-KEY("sleep*") OR TITLE-ABS-KEY("wake*") OR TITLE-ABS-KEY("waking") OR TITLE-ABS-KEY("awake"))  OR  (TITLE-ABS-KEY("actigraph*") OR TITLE-ABS-KEY("actimetr*") OR TITLE-ABS-KEY("acceleromet*") OR TITLE-ABS-KEY("polysomnograph*") OR TITLE-ABS-KEY("EEG") OR TITLE-ABS-KEY("electroencephalogram") OR TITLE-ABS-KEY("MSLT") OR TITLE-ABS-KEY("MWT") OR TITLE-ABS-KEY("fitbit") OR TITLE-ABS-KEY("dreem") OR TITLE-ABS-KEY("Oura ring") OR TITLE-ABS-KEY("Gen3") OR TITLE-ABS-KEY("Fitbit") OR TITLE-ABS-KEY("Mi band"))  OR  (TITLE-ABS-KEY("circadian") OR TITLE-ABS-KEY("chronotype*") OR TITLE-ABS-KEY("chronotherap*") OR TITLE-ABS-KEY("eveningness") OR TITLE-ABS-KEY("morningness") OR TITLE-ABS-KEY("evening type*") OR TITLE-ABS-KEY("morning type*") OR TITLE-ABS-KEY("bedtime*") OR TITLE-ABS-KEY("time to bed") OR TITLE-ABS-KEY("time in bed") OR TITLE-ABS-KEY("shuteye") OR TITLE-ABS-KEY("shut-eye") OR TITLE-ABS-KEY("lights off") OR TITLE-ABS-KEY("lights on") OR TITLE-ABS-KEY("shift work*") OR TITLE-ABS-KEY("shiftwork*") OR TITLE-ABS-KEY("shift" w/2 "schedule") OR TITLE-ABS-KEY("shift" w/2 "schedules") OR TITLE-ABS-KEY("shift" w/2 "scheduling") OR TITLE-ABS-KEY("shifting" w/2 "schedule") OR TITLE-ABS-KEY("shifting" w/2 "schedules") OR TITLE-ABS-KEY("shifting" w/2 "scheduling") OR TITLE-ABS-KEY("working" w/2 "hours") OR TITLE-ABS-KEY("work" w/2 "hours") OR TITLE-ABS-KEY("work" w/2 "schedule") OR TITLE-ABS-KEY("work" w/2 "schedules") OR TITLE-ABS-KEY("work" w/2 "scheduling") OR TITLE-ABS-KEY("working" w/2 "schedule") OR TITLE-ABS-KEY("working" w/2 "schedules") OR TITLE-ABS-KEY("jetlag") OR TITLE-ABS-KEY("jet-lag") OR TITLE-ABS-KEY("light*") OR TITLE-ABS-KEY("nois*") OR TITLE-ABS-KEY("WASO") OR TITLE-ABS-KEY("TIB") OR TITLE-ABS-KEY("SE"))  OR  (TITLE-ABS-KEY("fatigue*") OR TITLE-ABS-KEY("tired*") OR TITLE-ABS-KEY("somnolence") OR TITLE-ABS-KEY("nap") OR TITLE-ABS-KEY("napping") OR TITLE-ABS-KEY("alert*") OR TITLE-ABS-KEY("ESS") OR TITLE-ABS-KEY("KSS") OR TITLE-ABS-KEY("EDS") OR TITLE-ABS-KEY("day") OR TITLE-ABS-KEY("daytime") OR TITLE-ABS-KEY("night*") OR TITLE-ABS-KEY("drows*") OR TITLE-ABS-KEY("siesta"))  OR  (TITLE-ABS-KEY("insomnia") OR TITLE-ABS-KEY("restless legs syndrome") OR TITLE-ABS-KEY("restless leg syndrome") OR TITLE-ABS-KEY("Willis-Ekbom") OR TITLE-ABS-KEY("Wittmaack-Ekbom") OR TITLE-ABS-KEY("RLS") OR TITLE-ABS-KEY("periodic leg movement*") OR TITLE-ABS-KEY("periodic limb movement*") OR TITLE-ABS-KEY("snoring") OR TITLE-ABS-KEY("snore") OR TITLE-ABS-KEY("hypersomnia") OR TITLE-ABS-KEY("dyssomnia") OR TITLE-ABS-KEY("parasomnia") OR TITLE-ABS-KEY("narcolepsy") OR TITLE-ABS-KEY("night terror") OR TITLE-ABS-KEY("nightmare*") OR TITLE-ABS-KEY("apnea") OR TITLE-ABS-KEY("apnoea") OR TITLE-ABS-KEY("hypopnea") OR TITLE-ABS-KEY("hypopnea") OR TITLE-ABS-KEY("OSA") OR TITLE-ABS-KEY("OSAHS") OR TITLE-ABS-KEY("AHI") OR TITLE-ABS-KEY("CSA") OR TITLE-ABS-KEY("UARS") OR TITLE-ABS-KEY("upper airway resistance syndrome")) )  OR ( (TITLE-ABS-KEY("sleep*"))  AND  ( (TITLE-ABS-KEY("RU-SATED") OR TITLE-ABS-KEY("RU_SATED") OR TITLE-ABS-KEY("PSQI"))  OR  (TITLE-ABS-KEY("score*") OR TITLE-ABS-KEY("index") OR TITLE-ABS-KEY("indices") OR TITLE-ABS-KEY("multidimensional") OR TITLE-ABS-KEY("multi-dimensional") OR TITLE-ABS-KEY("multi*") OR TITLE-ABS-KEY("dimension*") OR TITLE-ABS-KEY("component*") OR TITLE-ABS-KEY("parameter*") OR TITLE-ABS-KEY("metric*") OR TITLE-ABS-KEY("composite") OR TITLE-ABS-KEY("combination*")) ) )  )) AND ((TITLE-ABS-KEY("mortality") OR TITLE-ABS-KEY("death*") OR TITLE-ABS-KEY("lethal")) OR ( (TITLE-ABS-KEY("SCD") OR TITLE-ABS-KEY("cardiopulmonary arrest") OR TITLE-ABS-KEY("cardiac arrest") OR TITLE-ABS-KEY("heart arrest") OR TITLE-ABS-KEY("asystole") OR TITLE-ABS-KEY("cardiac event*") OR TITLE-ABS-KEY("myocardial infarct*") OR TITLE-ABS-KEY("myocardial ischemia") OR TITLE-ABS-KEY("unstable angina") OR TITLE-ABS-KEY("acute coronary syndrome") OR TITLE-ABS-KEY("ACS") OR TITLE-ABS-KEY("AMI") OR TITLE-ABS-KEY("MI") OR TITLE-ABS-KEY("heart failure") OR TITLE-ABS-KEY("cardiac failure") OR TITLE-ABS-KEY("myocardial failure") OR TITLE-ABS-KEY("heart decompensation") OR TITLE-ABS-KEY("ventricular dysfunction") OR TITLE-ABS-KEY("CHF"))  OR  (TITLE-ABS-KEY("stroke") OR TITLE-ABS-KEY("cerebral infarct*") OR TITLE-ABS-KEY("cerebrovascular accident*") OR TITLE-ABS-KEY("CVA") OR TITLE-ABS-KEY("brain vascular accident*") OR TITLE-ABS-KEY("cerebrovascular apoplexy") OR TITLE-ABS-KEY("brain ischemia") OR TITLE-ABS-KEY("intracranial hemorrhage") OR TITLE-ABS-KEY("intracranial haemorrhage") OR TITLE-ABS-KEY("cerebral hemorrhage") OR TITLE-ABS-KEY("cerebral haemorrhage"))  OR  (TITLE-ABS-KEY("MACE") OR TITLE-ABS-KEY("major adverse cardiovascular event*") OR TITLE-ABS-KEY("infarct*")) )  ) |

**Abbreviations.** TITLE-ABS-KEY: search to be made in title and abstract fields, as well as keywords and controlled vocabulary from relevant indexed databases. **Clarifications.** 1) **bold font-weight** visually indicates blocks of search terms or parentheses encapsulating these; 2) **green color** denotes blocks of search terms; 3) dark blue color denotes search terms with the TITLE-ABS-KEY field setting without the proximity parameter; 4) light blue color denotes search terms with the TITLE-ABS-KEY field setting with the proximity parameter; 5) as Scopus does not have its own controlled vocabulary and the major ones (in indexed databases) are covered in the respective database, no specific search terms for controlled vocabularies were modified and added for this database.

## 8. Web of Science (including KCI, SciELO, and Web of Science Core Collection)

***Each database needs to be selected in the "DOCUMENTS" tab (options are visible to the right of "Search in:")***

| **#** | **Block name** | **Search terms** |
| --- | --- | --- |
| **1** | Sleep duration | **(**  (TS="sleep deprivation" OR TS="insufficient sleep")  OR  (TS=("quantity" NEAR/2 "sleep") OR TS=("quantities" NEAR/2 "sleep") OR TS=("amount" NEAR/2 "sleep") OR TS=("duration" NEAR/2 "sleep") OR TS=("length" NEAR/2 "sleep") OR TS=("time" NEAR/2 "sleep") OR TS=("period" NEAR/2 "sleep") OR TS=("hours" NEAR/2 "sleep") OR TS=("minutes" NEAR/2 "sleep") OR TS=("span" NEAR/2 "sleep"))  OR  (TS=("duration" NEAR/2 "asleep") OR TS=("length" NEAR/2 "asleep") OR TS=("time" NEAR/2 "asleep") OR TS=("period" NEAR/2 "asleep") OR TS=("hours" NEAR/2 "asleep") OR TS=("minutes" NEAR/2 "asleep"))  OR  (TS=("quantity" NEAR/2 "sleeping") OR TS=("quantities" NEAR/2 "sleeping") OR TS=("amount" NEAR/2 "sleeping") OR TS=("duration" NEAR/2 "sleeping") OR TS=("length" NEAR/2 "sleeping") OR TS=("time" NEAR/2 "sleeping") OR TS=("period" NEAR/2 "sleeping") OR TS=("hours" NEAR/2 "sleeping") OR TS=("minutes" NEAR/2 "sleeping") OR TS=("span" NEAR/2 "sleeping"))  OR  (TS=("short" NEAR/2 "sleep") OR TS=("long" NEAR/2 "sleep") OR TS=("extended" NEAR/2 "sleep"))  OR  (TS=("short" NEAR/2 "sleeper") OR TS=("long" NEAR/2 "sleeper") OR TS=("extended" NEAR/2 "sleeper"))  OR  (TS=("short" NEAR/2 "sleepers") OR TS=("long" NEAR/2 "sleepers") OR TS=("extended" NEAR/2 "sleepers"))  **)** |
| **2** | Sleep components | **(**  (TS="sleep*" OR TS="wake*" OR TS="waking" OR TS="awake")  OR  (TS="actigraph*" OR TS="actimetr*" OR TS="acceleromet*" OR TS="polysomnograph*" OR TS="EEG" OR TS="electroencephalogram" OR TS="MSLT" OR TS="MWT" OR TS="fitbit" OR TS="dreem" OR TS="Oura ring" OR TS="Gen3" OR TS="Fitbit" OR TS="Mi band")  OR  (TS="circadian" OR TS="chronotype*" OR TS="chronotherap*" OR TS="eveningness" OR TS="morningness" OR TS="evening type*" OR TS="morning type*" OR TS="bedtime*" OR TS="time to bed" OR TS="time in bed" OR TS="shuteye" OR TS="shut-eye" OR TS="lights off" OR TS="lights on" OR TS="shift work*" OR TS="shiftwork*" OR TS=("shift" NEAR/2 "schedule") OR TS=("shift" NEAR/2 "schedules") OR TS=("shift" NEAR/2 "scheduling") OR TS=("shifting" NEAR/2 "schedule") OR TS=("shifting" NEAR/2 "schedules") OR TS=("shifting" NEAR/2 "scheduling") OR TS=("working" NEAR/2 "hours") OR TS=("work" NEAR/2 "hours") OR TS=("work" NEAR/2 "schedule") OR TS=("work" NEAR/2 "schedules") OR TS=("work" NEAR/2 "scheduling") OR TS=("working" NEAR/2 "schedule") OR TS=("working" NEAR/2 "schedules") OR TS="jetlag" OR TS="jet-lag" OR TS="light*" OR TS="nois*" OR TS="WASO" OR TS="TIB" OR TS="SE")  OR  (TS="fatigue*" OR TS="tired*" OR TS="somnolence" OR TS="nap" OR TS="napping" OR TS="alert*" OR TS="ESS" OR TS="KSS" OR TS="EDS" OR TS="day" OR TS="daytime" OR TS="night*" OR TS="drows*" OR TS="siesta")  OR  (TS="insomnia" OR TS="restless legs syndrome" OR TS="restless leg syndrome" OR TS="Willis-Ekbom" OR TS="Wittmaack-Ekbom" OR TS="RLS" OR TS="periodic leg movement*" OR TS="periodic limb movement*" OR TS="snoring" OR TS="snore" OR TS="hypersomnia" OR TS="dyssomnia" OR TS="parasomnia" OR TS="narcolepsy" OR TS="night terror" OR TS="nightmare*" OR TS="apnea" OR TS="apnoea" OR TS="hypopnea" OR TS="hypopnea" OR TS="OSA" OR TS="OSAHS" OR TS="AHI" OR TS="CSA" OR TS="UARS" OR TS="upper airway resistance syndrome")  **)** |
| **3** | Multidimensionality | **(**  (TS="sleep*")  AND  (  (TS="RU-SATED" OR TS="RU_SATED" OR TS="PSQI")  OR  (TS="score*" OR TS="index" OR TS="indices" OR TS="multidimensional" OR TS="multi-dimensional" OR TS="multi*" OR TS="dimension*" OR TS="component*" OR TS="parameter*" OR TS="metric*" OR TS="composite" OR TS="combination*")  )  **)** |
| **4** | Multidimensional sleep health | **(#1** AND **(#2** OR **#3))** |
| **5** | All-cause outcomes | **(**TS="mortality" OR TS="death*" OR TS="lethal"**)** |
| **6** | Specific outcomes of interest | **(**  (TS="SCD" OR TS="cardiopulmonary arrest" OR TS="cardiac arrest" OR TS="heart arrest" OR TS="asystole" OR TS="cardiac event*" OR TS="myocardial infarct*" OR TS="myocardial ischemia" OR TS="unstable angina" OR TS="acute coronary syndrome" OR TS="ACS" OR TS="AMI" OR TS="MI" OR TS="heart failure" OR TS="cardiac failure" OR TS="myocardial failure" OR TS="heart decompensation" OR TS="ventricular dysfunction" OR TS="CHF")  OR  (TS="stroke" OR TS="cerebral infarct*" OR TS="cerebrovascular accident*" OR TS="CVA" OR TS="brain vascular accident*" OR TS="cerebrovascular apoplexy" OR TS="brain ischemia" OR TS="intracranial hemorrhage" OR TS="intracranial haemorrhage" OR TS="cerebral hemorrhage" OR TS="cerebral haemorrhage")  OR  (TS="MACE" OR TS="major adverse cardiovascular event*" OR TS="infarct*")  **)** |
| **7** | Outcomes | **(#5** OR **#6)** |
| **8** | Full query | **#4** AND **#7**  (( (TS="sleep deprivation" OR TS="insufficient sleep") OR (TS=("quantity" NEAR/2 "sleep") OR TS=("quantities" NEAR/2 "sleep") OR TS=("amount" NEAR/2 "sleep") OR TS=("duration" NEAR/2 "sleep") OR TS=("length" NEAR/2 "sleep") OR TS=("time" NEAR/2 "sleep") OR TS=("period" NEAR/2 "sleep") OR TS=("hours" NEAR/2 "sleep") OR TS=("minutes" NEAR/2 "sleep") OR TS=("span" NEAR/2 "sleep")) OR (TS=("duration" NEAR/2 "asleep") OR TS=("length" NEAR/2 "asleep") OR TS=("time" NEAR/2 "asleep") OR TS=("period" NEAR/2 "asleep") OR TS=("hours" NEAR/2 "asleep") OR TS=("minutes" NEAR/2 "asleep")) OR (TS=("quantity" NEAR/2 "sleeping") OR TS=("quantities" NEAR/2 "sleeping") OR TS=("amount" NEAR/2 "sleeping") OR TS=("duration" NEAR/2 "sleeping") OR TS=("length" NEAR/2 "sleeping") OR TS=("time" NEAR/2 "sleeping") OR TS=("period" NEAR/2 "sleeping") OR TS=("hours" NEAR/2 "sleeping") OR TS=("minutes" NEAR/2 "sleeping") OR TS=("span" NEAR/2 "sleeping")) OR (TS=("short" NEAR/2 "sleep") OR TS=("long" NEAR/2 "sleep") OR TS=("extended" NEAR/2 "sleep")) OR (TS=("short" NEAR/2 "sleeper") OR TS=("long" NEAR/2 "sleeper") OR TS=("extended" NEAR/2 "sleeper")) OR (TS=("short" NEAR/2 "sleepers") OR TS=("long" NEAR/2 "sleepers") OR TS=("extended" NEAR/2 "sleepers")) ) AND (( (TS="sleep*" OR TS="wake*" OR TS="waking" OR TS="awake") OR (TS="actigraph*" OR TS="actimetr*" OR TS="acceleromet*" OR TS="polysomnograph*" OR TS="EEG" OR TS="electroencephalogram" OR TS="MSLT" OR TS="MWT" OR TS="fitbit" OR TS="dreem" OR TS="Oura ring" OR TS="Gen3" OR TS="Fitbit" OR TS="Mi band") OR (TS="circadian" OR TS="chronotype*" OR TS="chronotherap*" OR TS="eveningness" OR TS="morningness" OR TS="evening type*" OR TS="morning type*" OR TS="bedtime*" OR TS="time to bed" OR TS="time in bed" OR TS="shuteye" OR TS="shut-eye" OR TS="lights off" OR TS="lights on" OR TS="shift work*" OR TS="shiftwork*" OR TS=("shift" NEAR/2 "schedule") OR TS=("shift" NEAR/2 "schedules") OR TS=("shift" NEAR/2 "scheduling") OR TS=("shifting" NEAR/2 "schedule") OR TS=("shifting" NEAR/2 "schedules") OR TS=("shifting" NEAR/2 "scheduling") OR TS=("working" NEAR/2 "hours") OR TS=("work" NEAR/2 "hours") OR TS=("work" NEAR/2 "schedule") OR TS=("work" NEAR/2 "schedules") OR TS=("work" NEAR/2 "scheduling") OR TS=("working" NEAR/2 "schedule") OR TS=("working" NEAR/2 "schedules") OR TS="jetlag" OR TS="jet-lag" OR TS="light*" OR TS="nois*" OR TS="WASO" OR TS="TIB" OR TS="SE") OR (TS="fatigue*" OR TS="tired*" OR TS="somnolence" OR TS="nap" OR TS="napping" OR TS="alert*" OR TS="ESS" OR TS="KSS" OR TS="EDS" OR TS="day" OR TS="daytime" OR TS="night*" OR TS="drows*" OR TS="siesta") OR (TS="insomnia" OR TS="restless legs syndrome" OR TS="restless leg syndrome" OR TS="Willis-Ekbom" OR TS="Wittmaack-Ekbom" OR TS="RLS" OR TS="periodic leg movement*" OR TS="periodic limb movement*" OR TS="snoring" OR TS="snore" OR TS="hypersomnia" OR TS="dyssomnia" OR TS="parasomnia" OR TS="narcolepsy" OR TS="night terror" OR TS="nightmare*" OR TS="apnea" OR TS="apnoea" OR TS="hypopnea" OR TS="hypopnea" OR TS="OSA" OR TS="OSAHS" OR TS="AHI" OR TS="CSA" OR TS="UARS" OR TS="upper airway resistance syndrome") ) OR ( (TS="sleep*") AND ( (TS="RU-SATED" OR TS="RU_SATED" OR TS="PSQI") OR (TS="score*" OR TS="index" OR TS="indices" OR TS="multidimensional" OR TS="multi-dimensional" OR TS="multi*" OR TS="dimension*" OR TS="component*" OR TS="parameter*" OR TS="metric*" OR TS="composite" OR TS="combination*") ) ) )) AND ((TS="mortality" OR TS="death*" OR TS="lethal") OR ( (TS="SCD" OR TS="cardiopulmonary arrest" OR TS="cardiac arrest" OR TS="heart arrest" OR TS="asystole" OR TS="cardiac event*" OR TS="myocardial infarct*" OR TS="myocardial ischemia" OR TS="unstable angina" OR TS="acute coronary syndrome" OR TS="ACS" OR TS="AMI" OR TS="MI" OR TS="heart failure" OR TS="cardiac failure" OR TS="myocardial failure" OR TS="heart decompensation" OR TS="ventricular dysfunction" OR TS="CHF") OR (TS="stroke" OR TS="cerebral infarct*" OR TS="cerebrovascular accident*" OR TS="CVA" OR TS="brain vascular accident*" OR TS="cerebrovascular apoplexy" OR TS="brain ischemia" OR TS="intracranial hemorrhage" OR TS="intracranial haemorrhage" OR TS="cerebral hemorrhage" OR TS="cerebral haemorrhage") OR (TS="MACE" OR TS="major adverse cardiovascular event*" OR TS="infarct*") ) ) |

**Abbreviations.** TS: search to be made in title, abstract, author keywords, and Keyword Plus^®^ fields. **Clarifications.** 1) **bold font-weight** visually indicates blocks of search terms or parentheses encapsulating these; 2) **green color** denotes blocks of search terms; 3) dark blue color denotes search terms with the TS field setting without the proximity parameter; 4) light blue color denotes search terms with the TS field setting with the proximity parameter; 5) as Web of Science does not have its own controlled vocabulary, no such search terms were defined for this database.

## 9. WHO Global Index Medicus (including AIM, IMEMR, IMSEAR, LILACS, and WPRIM)

| **#** | **Block name** | **Search terms** |
| --- | --- | --- |
| **1** | Sleep duration | **(**  (tw:"sleep deprivation" OR tw:"insufficient sleep")  OR  (tw:("quantity of sleep") OR tw:("sleep quantity") OR tw:("amount of sleep") OR tw:("sleep amount") OR tw:("duration of sleep") OR tw:("sleep duration") OR tw:("time of sleep") OR tw:("sleep time") OR tw:("period of sleep") OR tw:("sleep period") OR tw:("hours of sleep") OR tw:("sleep hours") OR tw:("minutes of sleep") OR tw:("span of sleep") OR tw:("sleep span"))  OR  (tw:("duration asleep") OR tw:("length asleep") OR tw:("time asleep") OR tw:("period asleep") OR tw:("hours asleep") OR tw:("minutes asleep"))  OR  (tw:("quantity of sleeping") OR tw:("sleeping quantity") OR tw:("quantities of sleeping") OR tw:("sleeping quantities") OR tw:("amount of sleeping") OR tw:("duration of sleeping") OR tw:("sleeping duration") OR tw:("length of sleeping") OR tw:("sleeping length") OR tw:("time sleeping") OR tw:("sleeping time") OR tw:("period sleeping") OR tw:("sleeping period") OR tw:("hours sleeping") OR tw:("sleeping hours") OR tw:("minutes of sleeping") OR tw:("sleeping span"))  OR  (tw:("short sleep") OR tw:("long sleep") OR tw:("extended sleep"))  OR  (tw:("short sleeper") OR tw:("long sleeper") OR tw:("extended sleeper"))  OR  (tw:("short sleepers") OR tw:("long sleepers") OR tw:("extended sleepers"))  **)** |
| **2** | Sleep components | **(**  (tw:"sleep*" OR tw:"wake*" OR tw:"waking" OR tw:"awake")  OR  (tw:"actigraph*" OR tw:"actimetr*" OR tw:"acceleromet*" OR tw:"polysomnograph*" OR tw:"EEG" OR tw:"electroencephalogram" OR tw:"MSLT" OR tw:"MWT" OR tw:"fitbit" OR tw:"dreem" OR tw:"Oura ring" OR tw:"Gen3" OR tw:"Fitbit" OR tw:"Mi band")  OR  (tw:"circadian" OR tw:"chronotype*" OR tw:"chronotherap*" OR tw:"eveningness" OR tw:"morningness" OR tw:"evening type*" OR tw:"morning type*" OR tw:"bedtime*" OR tw:"time to bed" OR tw:"time in bed" OR tw:"shuteye" OR tw:"shut-eye" OR tw:"lights off" OR tw:"lights on" OR tw:"shift work*" OR tw:"shiftwork*" OR ("shift schedule") OR tw:("shift schedules") OR tw:("shift scheduling") OR tw:("shift-scheduling") OR tw:("shifting of schedule") OR tw:("schedule shifting") OR tw:("shifting schedules") OR tw:("working hours") OR tw:("hours of work") OR tw:("work hours") OR tw:("work schedule") OR tw:("schedule of work") OR tw:("work schedules") OR tw:("schedules of work") OR tw:("work scheduling") OR tw:("scheduling of work") OR tw:("working schedule") OR tw:("schedule of working") OR tw:("working schedules") OR tw:("schedules of working") OR "jetlag" OR tw:"jet-lag" OR tw:"light*" OR tw:"nois*" OR tw:"WASO" OR tw:"TIB" OR tw:"SE")  OR  (tw:"fatigue*" OR tw:"tired*" OR tw:"somnolence" OR tw:"nap" OR tw:"napping" OR tw:"alert*" OR tw:"ESS" OR tw:"KSS" OR tw:"EDS" OR tw:"day" OR tw:"daytime" OR tw:"night*" OR tw:"drows*" OR tw:"siesta")  OR  (tw:"insomnia" OR tw:"restless legs syndrome" OR tw:"restless leg syndrome" OR tw:"Willis-Ekbom" OR tw:"Wittmaack-Ekbom" OR tw:"RLS" OR tw:"periodic leg movement*" OR tw:"periodic limb movement*" OR tw:"snoring" OR tw:"snore" OR tw:"hypersomnia" OR tw:"dyssomnia" OR tw:"parasomnia" OR tw:"narcolepsy" OR tw:"night terror" OR tw:"nightmare*" OR tw:"apnea" OR tw:"apnoea" OR tw:"hypopnea" OR tw:"hypopnea" OR tw:"OSA" OR tw:"OSAHS" OR tw:"AHI" OR tw:"CSA" OR tw:"UARS" OR tw:"upper airway resistance syndrome")  **)** |
| **3** | Multidimensionality | **(**  (tw:"sleep*")  AND  (  (tw:"RU-SATED" OR tw:"RU_SATED" OR tw:"PSQI")  OR  (tw:"score*" OR tw:"index" OR tw:"indices" OR tw:"multidimensional" OR tw:"multi-dimensional" OR tw:"multi*" OR tw:"dimension*" OR tw:"component*" OR tw:"parameter*" OR tw:"metric*" OR tw:"composite" OR tw:"combination*")  )  **)** |
| **4** | Multidimensional sleep health | **(#1** AND **(#2** OR **#3))** |
| **5** | All-cause outcomes | **(**tw:"mortality" OR tw:"death*" OR tw:"lethal"**)** |
| **6** | Specific outcomes of interest | **(**  (tw:"SCD" OR tw:"cardiopulmonary arrest" OR tw:"cardiac arrest" OR tw:"heart arrest" OR tw:"asystole" OR tw:"cardiac event*" OR tw:"myocardial infarct*" OR tw:"myocardial ischemia" OR tw:"unstable angina" OR tw:"acute coronary syndrome" OR tw:"ACS" OR tw:"AMI" OR tw:"MI" OR tw:"heart failure" OR tw:"cardiac failure" OR tw:"myocardial failure" OR tw:"heart decompensation" OR tw:"ventricular dysfunction" OR tw:"CHF")  OR  (tw:"stroke" OR tw:"cerebral infarct*" OR tw:"cerebrovascular accident*" OR tw:"CVA" OR tw:"brain vascular accident*" OR tw:"cerebrovascular apoplexy" OR tw:"brain ischemia" OR tw:"intracranial hemorrhage" OR tw:"intracranial haemorrhage" OR tw:"cerebral hemorrhage" OR tw:"cerebral haemorrhage")  OR  (tw:"MACE" OR tw:"major adverse cardiovascular event*" OR tw:"infarct*")  **)** |
| **7** | Outcomes | **(#5** OR **#6)** |
| **8** | Full query | **#4** AND **#7**  (**(** (tw:"sleep deprivation" OR tw:"insufficient sleep") OR (tw:("quantity of sleep") OR tw:("sleep quantity") OR tw:("amount of sleep") OR tw:("sleep amount") OR tw:("duration of sleep") OR tw:("sleep duration") OR tw:("time of sleep") OR tw:("sleep time") OR tw:("period of sleep") OR tw:("sleep period") OR tw:("hours of sleep") OR tw:("sleep hours") OR tw:("minutes of sleep") OR tw:("span of sleep") OR tw:("sleep span")) OR (tw:("duration asleep") OR tw:("length asleep") OR tw:("time asleep") OR tw:("period asleep") OR tw:("hours asleep") OR tw:("minutes asleep")) OR (tw:("quantity of sleeping") OR tw:("sleeping quantity") OR tw:("quantities of sleeping") OR tw:("sleeping quantities") OR tw:("amount of sleeping") OR tw:("duration of sleeping") OR tw:("sleeping duration") OR tw:("length of sleeping") OR tw:("sleeping length") OR tw:("time sleeping") OR tw:("sleeping time") OR tw:("period sleeping") OR tw:("sleeping period") OR tw:("hours sleeping") OR tw:("sleeping hours") OR tw:("minutes of sleeping") OR tw:("sleeping span")) OR (tw:("short sleep") OR tw:("long sleep") OR tw:("extended sleep")) OR (tw:("short sleeper") OR tw:("long sleeper") OR tw:("extended sleeper")) OR (tw:("short sleepers") OR tw:("long sleepers") OR tw:("extended sleepers")) **)** AND (**(** (tw:"sleep*" OR tw:"wake*" OR tw:"waking" OR tw:"awake") OR (tw:"actigraph*" OR tw:"actimetr*" OR tw:"acceleromet*" OR tw:"polysomnograph*" OR tw:"EEG" OR tw:"electroencephalogram" OR tw:"MSLT" OR tw:"MWT" OR tw:"fitbit" OR tw:"dreem" OR tw:"Oura ring" OR tw:"Gen3" OR tw:"Fitbit" OR tw:"Mi band") OR (tw:"circadian" OR tw:"chronotype*" OR tw:"chronotherap*" OR tw:"eveningness" OR tw:"morningness" OR tw:"evening type*" OR tw:"morning type*" OR tw:"bedtime*" OR tw:"time to bed" OR tw:"time in bed" OR tw:"shuteye" OR tw:"shut-eye" OR tw:"lights off" OR tw:"lights on" OR tw:"shift work*" OR tw:"shiftwork*" OR ("shift schedule") OR tw:("shift schedules") OR tw:("shift scheduling") OR tw:("shift-scheduling") OR tw:("shifting of schedule") OR tw:("schedule shifting") OR tw:("shifting schedules") OR tw:("working hours") OR tw:("hours of work") OR tw:("work hours") OR tw:("work schedule") OR tw:("schedule of work") OR tw:("work schedules") OR tw:("schedules of work") OR tw:("work scheduling") OR tw:("scheduling of work") OR tw:("working schedule") OR tw:("schedule of working") OR tw:("working schedules") OR tw:("schedules of working") OR "jetlag" OR tw:"jet-lag" OR tw:"light*" OR tw:"nois*" OR tw:"WASO" OR tw:"TIB" OR tw:"SE") OR (tw:"fatigue*" OR tw:"tired*" OR tw:"somnolence" OR tw:"nap" OR tw:"napping" OR tw:"alert*" OR tw:"ESS" OR tw:"KSS" OR tw:"EDS" OR tw:"day" OR tw:"daytime" OR tw:"night*" OR tw:"drows*" OR tw:"siesta") OR (tw:"insomnia" OR tw:"restless legs syndrome" OR tw:"restless leg syndrome" OR tw:"Willis-Ekbom" OR tw:"Wittmaack-Ekbom" OR tw:"RLS" OR tw:"periodic leg movement*" OR tw:"periodic limb movement*" OR tw:"snoring" OR tw:"snore" OR tw:"hypersomnia" OR tw:"dyssomnia" OR tw:"parasomnia" OR tw:"narcolepsy" OR tw:"night terror" OR tw:"nightmare*" OR tw:"apnea" OR tw:"apnoea" OR tw:"hypopnea" OR tw:"hypopnea" OR tw:"OSA" OR tw:"OSAHS" OR tw:"AHI" OR tw:"CSA" OR tw:"UARS" OR tw:"upper airway resistance syndrome") **) OR (** (tw:"sleep*") AND ( (tw:"RU-SATED" OR tw:"RU_SATED" OR tw:"PSQI") OR (tw:"score*" OR tw:"index" OR tw:"indices" OR tw:"multidimensional" OR tw:"multi-dimensional" OR tw:"multi*" OR tw:"dimension*" OR tw:"component*" OR tw:"parameter*" OR tw:"metric*" OR tw:"composite" OR tw:"combination*") ) **)**)) AND (**(**tw:"mortality" OR tw:"death*" OR tw:"lethal"**) OR (** (tw:"SCD" OR tw:"cardiopulmonary arrest" OR tw:"cardiac arrest" OR tw:"heart arrest" OR tw:"asystole" OR tw:"cardiac event*" OR tw:"myocardial infarct*" OR tw:"myocardial ischemia" OR tw:"unstable angina" OR tw:"acute coronary syndrome" OR tw:"ACS" OR tw:"AMI" OR tw:"MI" OR tw:"heart failure" OR tw:"cardiac failure" OR tw:"myocardial failure" OR tw:"heart decompensation" OR tw:"ventricular dysfunction" OR tw:"CHF") OR (tw:"stroke" OR tw:"cerebral infarct*" OR tw:"cerebrovascular accident*" OR tw:"CVA" OR tw:"brain vascular accident*" OR tw:"cerebrovascular apoplexy" OR tw:"brain ischemia" OR tw:"intracranial hemorrhage" OR tw:"intracranial haemorrhage" OR tw:"cerebral hemorrhage" OR tw:"cerebral haemorrhage") OR (tw:"MACE" OR tw:"major adverse cardiovascular event*" OR tw:"infarct*") **)**) |

**Abbreviations.** tw: search to be made in title, abstract, and Health Sciences Descriptors (DeCS)/Medical Subject Headings (MeSH) controlled vocabularies. **Clarifications.** 1) **bold font-weight** visually indicates blocks of search terms or parentheses encapsulating these; 2) **green color** denotes blocks of search terms; 3) dark blue color denotes search terms with the tw field setting.

## 10. WorldCat Dissertations and Theses

| **#** | **Block name** | **Search terms** |
| --- | --- | --- |
| **1** | Sleep duration | **(**  ("sleep deprivation" OR "insufficient sleep")  OR  (("quantity of sleep") OR ("sleep quantity") OR ("amount of sleep") OR ("sleep amount") OR ("duration of sleep") OR ("sleep duration") OR ("time of sleep") OR ("sleep time") OR ("period of sleep") OR ("sleep period") OR ("hours of sleep") OR ("sleep hours") OR ("minutes of sleep") OR ("span of sleep") OR ("sleep span"))  OR  (("duration asleep") OR ("length asleep") OR ("time asleep") OR ("period asleep") OR ("hours asleep") OR ("minutes asleep"))  OR  (("quantity of sleeping") OR ("sleeping quantity") OR ("quantities of sleeping") OR ("sleeping quantities") OR ("amount of sleeping") OR ("duration of sleeping") OR ("sleeping duration") OR ("length of sleeping") OR ("sleeping length") OR ("time sleeping") OR ("sleeping time") OR ("period sleeping") OR ("sleeping period") OR ("hours sleeping") OR ("sleeping hours") OR ("minutes of sleeping") OR ("sleeping span"))  OR  (("short sleep") OR ("long sleep") OR ("extended sleep"))  OR  (("short sleeper") OR ("long sleeper") OR ("extended sleeper"))  OR  (("short sleepers") OR ("long sleepers") OR ("extended sleepers"))  **)** |
| **2** | Sleep components | **(**  ("sleep*" OR "wake*" OR "waking" OR "awake")  OR  ("actigraph*" OR "actimetr*" OR "acceleromet*" OR "polysomnograph*" OR "EEG" OR "electroencephalogram" OR "MSLT" OR "MWT" OR "fitbit" OR "dreem" OR "Oura ring" OR "Gen3" OR "Fitbit" OR "Mi band")  OR  ("circadian" OR "chronotype*" OR "chronotherap*" OR "eveningness" OR "morningness" OR "evening type*" OR "morning type*" OR "bedtime*" OR "time to bed" OR "time in bed" OR "shuteye" OR "shut-eye" OR "lights off" OR "lights on" OR "shift work*" OR "shiftwork*" OR ("shift schedule") OR ("shift schedules") OR ("shift scheduling") OR ("shift-scheduling") OR ("shifting of schedule") OR ("schedule shifting") OR ("shifting schedules") OR ("working hours") OR ("hours of work") OR ("work hours") OR ("work schedule") OR ("schedule of work") OR ("work schedules") OR ("schedules of work") OR ("work scheduling") OR ("scheduling of work") OR ("working schedule") OR ("schedule of working") OR ("working schedules") OR ("schedules of working") OR "jetlag" OR "jet-lag" OR "light*" OR "nois*" OR "WASO" OR "TIB" OR "SE")  OR  ("fatigue*" OR "tired*" OR "somnolence" OR "nap" OR "napping" OR "alert*" OR "ESS" OR "KSS" OR "EDS" OR "day" OR "daytime" OR "night*" OR "drows*" OR "siesta")  OR  ("insomnia" OR "restless legs syndrome" OR "restless leg syndrome" OR "Willis-Ekbom" OR "Wittmaack-Ekbom" OR "RLS" OR "periodic leg movement*" OR "periodic limb movement*" OR "snoring" OR "snore" OR "hypersomnia" OR "dyssomnia" OR "parasomnia" OR "narcolepsy" OR "night terror" OR "nightmare*" OR "apnea" OR "apnoea" OR "hypopnea" OR "hypopnea" OR "OSA" OR "OSAHS" OR "AHI" OR "CSA" OR "UARS" OR "upper airway resistance syndrome")  **)** |
| **3** | Multidimensionality | **(**  ("sleep*")  AND  (  ("RU-SATED" OR "RU_SATED" OR "PSQI")  OR  ("score*" OR "index" OR "indices" OR "multidimensional" OR "multi-dimensional" OR "multi*" OR "dimension*" OR "component*" OR "parameter*" OR "metric*" OR "composite" OR "combination*")  )  **)** |
| **4** | Multidimensional sleep health | **(#1** AND **(#2** OR **#3))** |
| **5** | All-cause outcomes | **(**"mortality" OR "death*" OR "lethal"**)** |
| **6** | Specific outcomes of interest | **(**  ("SCD" OR "cardiopulmonary arrest" OR "cardiac arrest" OR "heart arrest" OR "asystole" OR "cardiac event*" OR "myocardial infarct*" OR "myocardial ischemia" OR "unstable angina" OR "acute coronary syndrome" OR "ACS" OR "AMI" OR "MI" OR "heart failure" OR "cardiac failure" OR "myocardial failure" OR "heart decompensation" OR "ventricular dysfunction" OR "CHF")  OR  ("stroke" OR "cerebral infarct*" OR "cerebrovascular accident*" OR "CVA" OR "brain vascular accident*" OR "cerebrovascular apoplexy" OR "brain ischemia" OR "intracranial hemorrhage" OR "intracranial haemorrhage" OR "cerebral hemorrhage" OR "cerebral haemorrhage")  OR  ("MACE" OR "major adverse cardiovascular event*" OR "infarct*")  **)** |
| **7** | Outcomes | **(#5** OR **#6)** |
| **8** | Full query | **#4** AND **#7**  (**(** ("sleep deprivation" OR "insufficient sleep") OR (("quantity of sleep") OR ("sleep quantity") OR ("amount of sleep") OR ("sleep amount") OR ("duration of sleep") OR ("sleep duration") OR ("time of sleep") OR ("sleep time") OR ("period of sleep") OR ("sleep period") OR ("hours of sleep") OR ("sleep hours") OR ("minutes of sleep") OR ("span of sleep") OR ("sleep span")) OR (("duration asleep") OR ("length asleep") OR ("time asleep") OR ("period asleep") OR ("hours asleep") OR ("minutes asleep")) OR (("quantity of sleeping") OR ("sleeping quantity") OR ("quantities of sleeping") OR ("sleeping quantities") OR ("amount of sleeping") OR ("duration of sleeping") OR ("sleeping duration") OR ("length of sleeping") OR ("sleeping length") OR ("time sleeping") OR ("sleeping time") OR ("period sleeping") OR ("sleeping period") OR ("hours sleeping") OR ("sleeping hours") OR ("minutes of sleeping") OR ("sleeping span")) OR (("short sleep") OR ("long sleep") OR ("extended sleep")) OR (("short sleeper") OR ("long sleeper") OR ("extended sleeper")) OR (("short sleepers") OR ("long sleepers") OR ("extended sleepers")) **)** AND (**(** ("sleep*" OR "wake*" OR "waking" OR "awake") OR ("actigraph*" OR "actimetr*" OR "acceleromet*" OR "polysomnograph*" OR "EEG" OR "electroencephalogram" OR "MSLT" OR "MWT" OR "fitbit" OR "dreem" OR "Oura ring" OR "Gen3" OR "Fitbit" OR "Mi band") OR ("circadian" OR "chronotype*" OR "chronotherap*" OR "eveningness" OR "morningness" OR "evening type*" OR "morning type*" OR "bedtime*" OR "time to bed" OR "time in bed" OR "shuteye" OR "shut-eye" OR "lights off" OR "lights on" OR "shift work*" OR "shiftwork*" OR ("shift schedule") OR ("shift schedules") OR ("shift scheduling") OR ("shift-scheduling") OR ("shifting of schedule") OR ("schedule shifting") OR ("shifting schedules") OR ("working hours") OR ("hours of work") OR ("work hours") OR ("work schedule") OR ("schedule of work") OR ("work schedules") OR ("schedules of work") OR ("work scheduling") OR ("scheduling of work") OR ("working schedule") OR ("schedule of working") OR ("working schedules") OR ("schedules of working") OR "jetlag" OR "jet-lag" OR "light*" OR "nois*" OR "WASO" OR "TIB" OR "SE") OR ("fatigue*" OR "tired*" OR "somnolence" OR "nap" OR "napping" OR "alert*" OR "ESS" OR "KSS" OR "EDS" OR "day" OR "daytime" OR "night*" OR "drows*" OR "siesta") OR ("insomnia" OR "restless legs syndrome" OR "restless leg syndrome" OR "Willis-Ekbom" OR "Wittmaack-Ekbom" OR "RLS" OR "periodic leg movement*" OR "periodic limb movement*" OR "snoring" OR "snore" OR "hypersomnia" OR "dyssomnia" OR "parasomnia" OR "narcolepsy" OR "night terror" OR "nightmare*" OR "apnea" OR "apnoea" OR "hypopnea" OR "hypopnea" OR "OSA" OR "OSAHS" OR "AHI" OR "CSA" OR "UARS" OR "upper airway resistance syndrome") **) OR (** ("sleep*") AND ( ("RU-SATED" OR "RU_SATED" OR "PSQI") OR ("score*" OR "index" OR "indices" OR "multidimensional" OR "multi-dimensional" OR "multi*" OR "dimension*" OR "component*" OR "parameter*" OR "metric*" OR "composite" OR "combination*") ) **)**)) AND (**(**"mortality" OR "death*" OR "lethal"**) OR (** ("SCD" OR "cardiopulmonary arrest" OR "cardiac arrest" OR "heart arrest" OR "asystole" OR "cardiac event*" OR "myocardial infarct*" OR "myocardial ischemia" OR "unstable angina" OR "acute coronary syndrome" OR "ACS" OR "AMI" OR "MI" OR "heart failure" OR "cardiac failure" OR "myocardial failure" OR "heart decompensation" OR "ventricular dysfunction" OR "CHF") OR ("stroke" OR "cerebral infarct*" OR "cerebrovascular accident*" OR "CVA" OR "brain vascular accident*" OR "cerebrovascular apoplexy" OR "brain ischemia" OR "intracranial hemorrhage" OR "intracranial haemorrhage" OR "cerebral hemorrhage" OR "cerebral haemorrhage") OR ("MACE" OR "major adverse cardiovascular event*" OR "infarct*") **)**) |
